# Supplementary material for: Retrospective cohort study evaluating clinical, biochemical and pharmacological prognostic factors for prostate cancer progression using primary care data
Source: BMJ Open. 2021 Feb 12;11(2):e044420. doi: 10.1136/bmjopen-2020-044420 (PMC7883851; doi:10.1136/bmjopen-2020-044420)
Supplement: Supplementary data [file bmjopen-2020-044420supp001.pdf]

n = 10,901			Prostate cancer mortality			All-cause mortality			Systemic therapy		
**Factor**	**Mean (SD)**	**Missing [n (%)]**	**HR per SD**	**95% CI**	**p**	**HR per SD**	**95% CI**	**p**	**HR per SD**	**95% CI**	**p**
Age	74.39 (9.03)	0	1.66	1.56, 1.76	<0.01	1.82	1.76, 1.89	<0.01	0.81	0.77, 0.84	<0.01
BMI	27.43 (4.48)	394 (3.61%)	0.96	0.91, 1.02	0.19	0.97	0.94, 1.01	0.15	1.07	1.03, 1.12	<0.01
Triglycerides	1.45 (0.80)	3,856 (35.37%)	0.93	0.84, 1.02	0.12	0.97	0.92, 1.02	0.28	0.99	0.95, 1.04	0.82
HDL cholesterol	1.35 (0.43)	3,954 (36.27%)	0.98	0.88, 1.09	0.66	0.98	0.92, 1.04	0.49	1.03	0.98, 1.08	0.23
LDL cholesterol	2.95 (0.99)	4,698 (43.10%)	0.90	0.81, 0.99	0.03	0.87	0.82, 0.92	<0.01	0.95	0.91, 0.99	0.03
Hb	144.28 (14.35)	2,696 (24.73%)	0.55	0.51, 0.58	<0.01	0.62	0.59, 0.64	<0.01	0.95	0.89, 0.99	0.03
Albumin	41.83 (3.94)	2,954 (27.10%)	0.71	0.67, 0.74	<0.01	0.74	0.71, 0.77	<0.01	0.96	0.91, 1.00	0.06
Random glucose	5.70 (2.11)	4,525 (41.51%)	1.18	1.10, 1.27	<0.01	1.14	1.09, 1.20	<0.01	1.02	0.97, 1.07	0.48
	**Median (IQR)**	**Missing [n (%)]**									
PSA	8.4 (5.55, 14.60)	2,352 (21.58%)	1.45	1.39, 1.51	<0.01	1.36	1.31, 1.41	<0.01	1.01	0.86, 1.18	0.90
CRP	3.9 (2, 8)	8,061 (73.95%)	1.27	1.19, 1.36	<0.01	1.22	1.16, 1.28	<0.01	1.01	0.92, 1.10	0.90
Ferritin	108.6 (47, 196)	9,495 (87.10%)	1.48	1.25, 1.75	<0.01	1.13	0.97, 1.31	0.11	1.02	0.89, 1.16	0.79

Table S1 – Unadjusted HRs for prognostic factors for men with localised disease associated with outcomes

HR – Hazard Ratio; SD – Standard Deviation; BMI – Body Mass Index; PSA – Prostate Specific Antigen; HDL – High Density Lipoprotein; LDL – Low Density Lipoprotein; HbA1c – Haemoglobin A1c; CRP – C-Reactive Protein; Hb – Haemoglobin

n = 10,901			Prostate cancer mortality			All-cause mortality			Systemic therapy		
**Factor**	**n (%)**	**Missing [n(%)]**	**HR**	**95% CI**	**p**	**HR**	**95% CI**	**p**	**HR**	**95% CI**	**p**
Smoker (current/ ex-)	5,112 (46.89%)	777 (7.13%)	1.24	1.09, 1.40	<0.01	1.36	1.26, 1.46	<0.01	1.20	1.10, 1.30	<0.01
Excess alcohol	1,829 (16.78%)	4,370 (40.09%)	0.75	0.62, 0.91	<0.01	0.67	0.60, 0.74	<0.01	0.92	0.80, 1.06	0.26
BPH	1,169 (10.72%)	3,526 (32.35%)	0.61	0.49, 0.76	<0.01	0.79	0.70, 0.88	<0.01	0.70	0.61, 0.80	<0.01
COPD	862 (7.91%)	3,583 (32.87%)	0.95	0.77, 1.18	0.67	1.41	1.26, 1.58	<0.01	1.08	0.94, 1.25	0.26
CVA	553 (5.07%)	3,584 (32.88%)	1.48	1.18, 1.85	<0.01	1.89	1.67, 2.13	<0.01	0.78	0.64, 0.96	0.02
IHD	1,548 (14.20%)	3,405 (31.24%)	1.62	1.39, 1.88	<0.01	1.79	1.64, 1.95	<0.01	0.89	0.79, 1.00	0.05
PVD	202 (1.85%)	3,582 (32.86%)	1.80	1.31, 2.49	<0.01	1.87	1.55, 2.25	<0.01	0.84	0.62, 1.14	0.26
T2DM	1,508 (13.83%)	3,448 (31.63%)	1.13	0.96, 1.33	0.16	1.03	0.94, 1.14	0.49	0.93	0.83, 1.04	0.20
Aspirin	426 (3.91%)	16 (0.15%)	2.33	1.88, 2.90	<0.01	2.18	1.90, 2.49	<0.01	1.24	0.99, 1.54	0.06
Metformin	33 (0.30%)		1.99	0.90, 4.46	0.09	2.63	1.71, 4.04	<0.01	2.59	1.47, 4.57	<0.01
Alpha blockers	305 (2.80%)		1.24	0.92, 1.67	0.16	1.17	0.97, 1.41	0.09	0.51	0.37, 0.71	<0.01
Beta blockers	265 (2.43%)		2.15	1.65, 2.81	<0.01	1.76	1.48, 2.10	<0.01	1.11	0.85, 1.46	0.43
Statins	339 (3.11%)		1.34	1.01, 1.77	0.04	0.93	0.76, 1.13	0.45	1.42	1.16, 1.73	<0.01
Vitamin D	465 (4.27%)		1.63	1.29, 2.05	<0.01	1.24	1.06, 1.46	<0.01	1.52	1.27, 1.82	<0.01

Table S2 – Unadjusted HRs for prognostic factors for men with localised disease associated with outcomes

HR – Hazard Ratio; BPH – Benign Prostatic Hypertrophy; COPD – Chronic Obstructive Pulmonary Disease; CVA – Cerebrovascular Accident; IHD – Ischaemic Heart Disease; PVD – Peripheral Vascular Disease; T2DM – Type 2 Diabetes Mellitus

**n = 42, 182**			**Prostate cancer mortality**			**All-cause mortality**			**Systemic therapy**		
**Factor**	**Mean (SD)**	**Missing [n (%)]**	**HR per SD**	**95% CI**	**p**	**HR per SD**	**95% CI**	**p**	**HR per SD**	**95% CI**	**p**
Age	76.70 (9.42)	0	1.55	1.49, 1.62	<0.01	1.78	1.74, 1.82	<0.01	0.67	0.65, 0.70	<0.01
BMI	27.37 (4.49)	394 (3.61%)	0.96	0.92, 0.99	0.05	0.96	0.94, 0.98	<0.01	1.06	1.02, 1.10	<0.01
Triglycerides	1.48 (0.82)	3,856 (35.37%)	0.92	0.87, 0.99	0.02	0.95	0.91, 0.98	<0.01	0.96	0.91, 1.00	0.06
HDL cholesterol	1.34 (0.53)	3,954 (36.27%)	1	0.94, 1.07	0.60	1	0.97, 1.04	0.80	1.03	0.99, 1.06	0.12
LDL cholesterol	2.84 (0.99)	4,698 (43.10%)	0.93	0.86, 0.99	0.03	0.89	0.86, 0.93	<0.01	1.05	1, 1.10	0.03
Hb	141.81 (16.58)	2,696 (24.73%)	0.61	0.58, 0.63	<0.01	0.66	0.64, 0.67	<0.01	1.07	1.02, 1.13	<0.01
Albumin	41.41 (4.31)	2,954 (27.10%)	1.71	0.99, 2.96	0.06	1.33	0.91, 1.94	0.15	0.75	0.46, 1.21	0.24
Random glucose	5.92 (2.11)	4,525 (41.51%)	1.06	1.01, 1.10	0.01	1.07	1.04, 1.09	<0.01	0.92	0.87, 0.98	0.01
	**Median (IQR)**	**Missing [n (%)]**									
PSA	10.4 (6.11, 24.6)	2,352 (21.58%)	1.28	0.96, 1.71	0.10	0.84	0.73, 0.98	0.02	2.37	1.88, 2.99	<0.01
CRP	5 (2, 10)	8,061 (73.95%)	2.50	1.03, 6.08	0.04	2.55	1.47, 4.42	<0.01	1.03	0.51, 2.06	0.94
Ferritin	101.5 (45, 197)	9,495 (87.10%)	1.10	1.06, 1.15	<0.01	1.04	0.99, 1.09	0.12	0.99	0.90, 1.09	.089

Table S3 – Unadjusted HRs for prognostic factors for men with localised disease and unknown location associated with outcomes

HR – Hazard Ratio; SD – Standard Deviation; BMI – Body Mass Index; PSA – Prostate Specific Antigen; HDL – High Density Lipoprotein; LDL – Low Density Lipoprotein; HbA1c – Haemoglobin A1c; CRP – C-Reactive Protein; Hb – Haemoglobin

**n = 42, 182**			**Prostate cancer mortality**			**All-cause mortality**			**Systemic therapy**		
**Factor**	**n (%)**	**Missing [n(%)]**	**HR**	**95% CI**	**p**	**HR**	**95% CI**	**p**	**HR**	**95% CI**	**p**
Smoker (current/ ex-)	19,215 (45.56%)	777 (7.13%)	1.24	1.09, 1.40	<0.01	1.36	1.26, 1.46	<0.01	1.20	1.10, 1.30	<0.01
Excess alcohol	5,926 (14.05%)	4,370 (40.09%)	0.75	0.62, 0.90	<0.01	0.79[b]	0.71, 0.88	<0.01	1.01	0.90, 1.13	0.86
BPH	4,318 (10.24%)	3,526 (32.35%)	0.61	0.49, 0.76	<0.01	0.79	0.70, 0.89	<0.01	0.70	0.61, 0.80	<0.01
COPD	3,866 (9.17%)	3,583 (32.87%)	0.95	0.77, 1.19	0.66	1.41	1.26, 1.58	<0.01	1.08	0.94, 1.25	0.26
CVA	2,973 (7.05%)	3,584 (32.88%)	1.48	1.19, 1.85	<0.01	1.89	1.67, 2.12	<0.01	0.78	0.64, 0.96	0.02
IHD	7,512 (17.81%)	3,405 (31.24%)	1.62	1.39, 1.88	<0.01	1.79	1.64, 1.95	<0.01	0.89	0.79, 1.00	0.05
PVD	1,138 (2.70%)	3,582 (32.86%)	1.8	1.31, 2.49	<0.01	1.89	1.55, 2.25	<0.01	0.84	0.62, 1.14	0.26
T2DM	6,233 (14.78%)	3,448 (31.63%)	1.13	0.96, 1.33	0.16	1.04	0.94, 1.14	0.49	0.93	0.83, 1.04	0.20
Aspirin	2,022 (4.79%)	16 (0.15%)	2.33	1.88, 2.90	<0.01	2.18	1.90, 2.49	<0.01	1.24	0.99, 1.54	0.06
Metformin	220 (0.52%)		1.99	0.90, 4.46	0.09	2.63	1.71, 4.04	<0.01	2.59	1.47, 4.57	<0.01
Alpha blockers	1,025 (2.43%)		1.24	0.92, 1.67	0.16	1.17	0.98, 1.41	0.09	0.51	0.37, 0.71	<0.01
Beta blockers	1,127 (2.67%)		2.15	1.65, 2.81	<0.01	1.76	1.48, 2.10	<0.01	1.11	0.85, 1.46	0.43
Statins	1,299 (3.08%)		1.34	1.01, 1.77	0.04	0.93	0.78, 1.13	0.45	1.42	1.16, 1.73	<0.01
Vitamin D	2,093 (4.96%)		1.63	1.29, 2.05	<0.01	1.24	1.06, 1.46	0.01	1.52	1.27, 1.88	<0.01

Table S4 – Unadjusted HRs for prognostic factors for men with localised disease and unknown location associated with outcomes

HR – Hazard Ratio; BPH – Benign Prostatic Hypertrophy; COPD – Chronic Obstructive Pulmonary Disease; CVA – Cerebrovascular Accident; IHD – Ischaemic Heart Disease;

PVD – Peripheral Vascular Disease; T2DM – Type 2 Diabetes Mellitus

n = 42, 182			Prostate cancer mortality			All-cause mortality			Systemic therapy		
**Factor**	**Mean (SD)**	**Missing [n (%)]**	**HR per SD[a]**	**95% CI**	**p**	**HR per SD[a]**	**95% CI**	**p**	**HR per SD[a]**	**95% CI**	**p**
Age	76.70 (9.42)	0	1.82	1.54, 2.16	<0.01	2.00	1.84, 2.19	<0.01	0.96	0.90, 1.01	0.01
BMI	27.37 (4.49)	394 (3.61%)	1.07	0.93, 1.23	0.36	0.96	0.89, 1.03	0.28	1.04	0.99, 1.09	0.10
Triglycerides	1.48 (0.82)	3,856 (35.37%)	0.89	0.70, 1.12	0.31	1.02[b]	0.92, 1.13	0.72	1.03	0.97, 1.09	0.38
HDL cholesterol	1.34 (0.53)	3,954 (36.27%)	1.04	0.88, 1.23	0.67	0.97[b]	0.86, 1.09	0.58	1.01	0.95, 1.07	0.79
LDL cholesterol	2.84 (0.99)	4,698 (43.10%)	0.84	0.68, 1.03	0.09	0.90[b]	0.82, 1.01	0.05	0.99	0.94, 1.05	0.81
Hb	141.81 (16.58)	2,696 (24.73%)	0.71	0.59, 0.85	<0.01	0.74	0.67, 0.80	<0.01	0.90	0.85, 0.96	<0.01
Albumin	41.41 (4.31)	2,954 (27.10%)	0.76	0.65, 0.89	<0.01	0.81	0.75, 0.88	<0.01	0.92	0.87, 0.97	<0.01
Random glucose	5.92 (2.11)	4,525 (41.51%)	1.28	1.08, 1.53	<0.01	1.11	0.99, 1.24	0.07	1.02[b]	0.95, 1.09	0.58
	**Median (IQR)**	**Missing [n (%)]**									
PSA	10.4 (6.11, 24.6)	2,352 (21.58%)	1.19	1.04, 1.35	0.01	1.14	1.02, 1.28	0.02	1.09	0.92, 1.30	0.33
CRP	5 (2, 10)	8,061 (73.95%)	1.41 [b]	1.12, 1.77	<0.01	1.28[b]	1.11, 1.47	<0.01	1.07	0.95, 1.20	0.25
Ferritin	101.5 (45, 197)	9,495 (87.10%)	1.80	1.08, 3.00	0.02	1.02[b]	0.67, 1.56	0.92	1.06	0.85, 1.32	0.62

Table S5 – Prognostic factors for men with localised disease and unknown location associated with outcomes

[a] Adjusted for age, PSA, Gleason score, TNM stage

[b] Proportional Hazards assumption test not met

HR – Hazard Ratio; SD – Standard Deviation; BMI – Body Mass Index; PSA – Prostate Specific Antigen; HDL – High Density Lipoprotein; LDL – Low Density Lipoprotein; HbA1c – Haemoglobin A1c; CRP – C-Reactive Protein; Hb – Haemoglobin

n = 42, 182			Prostate cancer mortality			All-cause mortality			Systemic therapy		
**Factor**	**n (%)**	**Missing [n(%)]**	**HR[a]**	**95% CI**	**p**	**HR[a]**	**95% CI**	**p**	**HR[a]**	**95% CI**	**p**
Smoker (current/ ex-)	19,215 (45.56%)	777 (7.13%)	1.41	1.04, 1.90	0.03	1.67	1.43, 1.96	<0.01	1.22	1.10, 1.34	<0.01
Excess alcohol	5,926 (14.05%)	4,370 (40.09%)	0.65	0.40, 1.05	0.08	0.89[b]	0.71, 1.13	0.35	0.99	0.86, 1.13	0.85
BPH	4,318 (10.24%)	3,526 (32.35%)	0.63	0.38, 1.05	0.08	0.82	0.65, 1.03	0.08	0.73	0.62, 0.86	<0.01
COPD	3,866 (9.17%)	3,583 (32.87%)	0.99	0.58, 1.66	0.96	1.68	1.35, 2.09	<0.01	1.18	0.99, 1.40	0.06
CVA	2,973 (7.05%)	3,584 (32.88%)	0.64	0.30, 1.38	0.26	1.47	1.12, 1.93	<0.01	0.90	0.71, 1.15	0.41
IHD	7,512 (17.81%)	3,405 (31.24%)	1.70	1.19, 2.44	<0.01	1.31	1.08, 1.58	<0.01	1.01	0.87, 1.00	0.9
PVD	1,138 (2.70%)	3,582 (32.86%)	2.52	1.23, 5.16	0.01	1.91	1.27, 2.85	<0.01	1.09	0.75, 1.59	0.64
T2DM	6,233 (14.78%)	3,448 (31.63%)	1.06	0.72, 1.58	0.76	0.97	0.79, 1.19	0.75	1.02	0.88, 1.17	0.83
Aspirin	2,022 (4.79%)	16 (0.15%)	1.55	0.79, 3.02	0.20	1.41	0.99, 2.00	0.06	1.23	0.95, 1.60	0.12
Metformin	220 (0.52%)					2.76	1.03, 7.38	0.04	1.43	0.64, 3.20	0.38
Alpha blockers	1,025 (2.43%)		1.28	0.59, 2.75	0.53	1.19	0.85, 1.67	0.32	0.55	0.38, 0.79	<0.01
Beta blockers	1,127 (2.67%)		1.76	0.82, 3.75	0.15	1.82	1.27, 2.62	<0.01	1.43	1.06, 1.93	0.02
Statins	1,299 (3.08%)		1.42	0.75, 2.69	0.28	0.87	0.57, 1.32	0.51	1.09	0.86, 1.37	0.48
Vitamin D	2,093 (4.96%)		1.13	0.55, 2.30	0.74	1.13	0.78, 1.65	0.51	1.38	1.12, 1.72	<0.01

Table S6 – Prognostic factors for men with localised disease and unknown location associated with outcomes

[a] Adjusted for age, PSA, Gleason score, TNM stage

[b] Proportional Hazards assumption test not met

HR – Hazard Ratio; BPH – Benign Prostatic Hypertrophy; COPD – Chronic Obstructive Pulmonary Disease; CVA – Cerebrovascular Accident; IHD – Ischaemic Heart Disease; PVD – Peripheral Vascular Disease; T2DM – Type 2 Diabetes Mellitus
