# Supplementary material for: Retrospective cohort study evaluating clinical, biochemical and pharmacological prognostic factors for prostate cancer progression using primary care data
Source: BMJ Open. 2021 Feb 12;11(2):e044420. doi: 10.1136/bmjopen-2020-044420 (PMC7883851; doi:10.1136/bmjopen-2020-044420)
Supplement: Author's manuscript [file bmjopen-2020-044420.draft_revisions.pdf]

BMJ Open

BMJ Open is committed to open peer review. As part of this commitment we make the peer review history of every article we publish publicly available.

When an article is published we post the peer reviewers' comments and the authors' responses online. We also post the versions of the paper that were used during peer review. These are the versions that the peer review comments apply to.

The versions of the paper that follow are the versions that were submitted during the peer review process. They are not the versions of record or the final published versions. They should not be cited or distributed as the published version of this manuscript.

BMJ Open is an open access journal and the full, final, typeset and author-corrected version of record of the manuscript is available on our site with no access controls, subscription charges or pay-per-view fees (http://bmjopen.bmj.com).

If you have any questions on BMJ Open's open peer review process please email info.bmjopen@bmj.com

BMJ Open

# Retrospective cohort study evaluating clinical, biochemical and pharmacological prognostic factors for prostate cancer progression using primary care data

| | |
|---|---|
| Journal: | *BMJ Open* |
| Manuscript ID | bmjopen-2020-044420 |
| Article Type: | Original research |
| Date Submitted by the Author: | 06-Sep-2020 |
| Complete List of Authors: | Merriel, Samuel; University of Exeter, College of Medicine & Health<br>Ingle, Suzanne; University of Bristol School of Social and Community Medicine, Centre for Academic Primary Care<br>May, Margaret; University of Bristol School of Social and Community Medicine,<br>Martin, Richard; University of Bristol, Social Medicine |
| Keywords: | Prostate disease < UROLOGY, Epidemiology < ONCOLOGY, PRIMARY CARE |
| | |

SCHOLARONE™
Manuscripts

*I, the Submitting Author has the right to grant and does grant on behalf of all authors of the Work (as defined in the below author licence), an exclusive licence and/or a non-exclusive licence for contributions from authors who are: i) UK Crown employees; ii) where BMJ has agreed a CC-BY licence shall apply, and/or iii) in accordance with the terms applicable for US Federal Government officers or employees acting as part of their official duties; on a worldwide, perpetual, irrevocable, royalty-free basis to BMJ Publishing Group Ltd ("BMJ") its licensees and where the relevant Journal is co-owned by BMJ to the co-owners of the Journal, to publish the Work in this journal and any other BMJ products and to exploit all rights, as set out in our licence.*

*The Submitting Author accepts and understands that any supply made under these terms is made by BMJ to the Submitting Author unless you are acting as an employee on behalf of your employer or a postgraduate student of an affiliated institution which is paying any applicable article publishing charge ("APC") for Open Access articles. Where the Submitting Author wishes to make the Work available on an Open Access basis (and intends to pay the relevant APC), the terms of reuse of such Open Access shall be governed by a Creative Commons licence – details of these licences and which Creative Commons licence will apply to this Work are set out in our licence referred to above.*

*Other than as permitted in any relevant BMJ Author's Self Archiving Policies, I confirm this Work has not been accepted for publication elsewhere, is not being considered for publication elsewhere and does not duplicate material already published. I confirm all authors consent to publication of this Work and authorise the granting of this licence.*

For peer review only

# Retrospective cohort study evaluating clinical, biochemical and pharmacological prognostic factors for prostate cancer progression using primary care data

Samuel W D Merriel[1, 2]
Suzanne M Ingle[2]
Margaret T May[2, 3]
Richard M Martin[2, 3]

1 Exeter Medical School, University of Exeter, Exeter, UK
2 Bristol Medical School, Population Health Sciences, University of Bristol, Bristol, UK
3 National Institute for Health Research (NIHR) Bristol Biomedical Research Centre, University Hospitals Bristol NHS Foundation Trust and the University of Bristol, Bristol, UK

**Corresponding author** – Samuel W D Merriel
1.18 College House, Heavitree Road, University of Exeter, Exeter, UK EX1 2LU
+441392 726002
s.w.d.merriel@exeter.ac.uk

For peer review only

# Abstract

**Objectives** – To confirm the association of previously reported prognostic factors with future progression of localised prostate cancer using primary care data and identify new potential prognostic factors for further assessment in prognostic model development and validation.

**Design** – Retrospective cohort study, employing Cox proportional hazards regression controlling for age, PSA, and Gleason score, stratified by diagnostic stage.

**Setting** – Primary care

**Participants** – Males with localised prostate cancer diagnosed between 01/01/1987 and 31/12/2016 within the Clinical Practice Research Datalink database, with linked data from the National Cancer Registration and Analysis Service and Office for National Statistics.

**Primary and Secondary outcomes** – Primary outcome measure was prostate cancer mortality. Secondary outcomes measures were all-cause mortality and commencing systematic therapy. Up-staging after diagnosis was not used as a secondary outcome in the final analysis owing to significant missing data.

**Results**

10,901 males (mean age 74.38 +/- 9.03 years) with localised prostate cancer were followed up for a mean of 14.12 (+/- 6.36) years. 2,331 (21.38%) men underwent systemic therapy and 3,250 (31.65%) died, including 1,250 (11.47%) from prostate cancer. Factors associated with an increased risk of prostate cancer mortality included age; high PSA; current or ex-smoker; ischaemic heart disease; high C-Reactive Protein; high ferritin; low haemoglobin; high blood glucose; and low albumin.

**Conclusions**

This study identified several new potential prognostic factors for prostate cancer progression, as well as confirming some known prognostic factors, in an independent primary care data set. Further research is needed to develop and validate a prognostic model for prostate cancer progression.


For peer review only

# Article summary

**Strengths and limitations of this study**

- Large retrospective cohort study of men with localised prostate cancer
- Mean follow-up 14.12 years
- Data available on a wide range of potential prognostic factors for prostate cancer progression
- Missing cancer stage and grade data from NCRAS cancer registry excluded a proportion of the cohort

For peer review only

# Main text

## Introduction

Prostate cancer prognosis and treatment decisions remain a challenging clinical area for clinicians and patients, particularly for men with localised disease at the time of diagnosis. In recent decades, prostate cancer detection rates in many countries have increased markedly, in part as a result of the rising use of asymptomatic prostate specific antigen (PSA) testing[1]. However, more intensive PSA-based detection of prostate cancer has not been convincingly directly correlated with reductions in prostate cancer mortality for all men[2], implying increasing over-detection of clinically insignificant tumours[3]. Treatments for prostate cancer carry a significant risk of morbidity for men[4,5], underlining the importance of being able to identify which men with tumours confined to the prostate at diagnosis are at higher risk of prostate cancer progression and mortality to inform discussions about management options.

Defining and measuring cancer progression with respect to treatment studies is outlined in the Response Evaluation Criteria in Solid Tumours (RECIST) criteria, which was originally published by the World Health Organisation in 2000[6] and most recently updated in 2009[7]. Evidence of tumour shrinkage on imaging and time to development of disease progression are used to measure treatment response. Definitions of cancer progression that are relevant to prognostic studies are less well defined, and numerous clinical, biological and surrogate markers of progression have been proposed in various studies. Prostate cancer mortality appears to be the logical ultimate endpoint of prostate cancer progression, but other measures such as development of metastases[8], biochemical recurrence[9], commencing systemic therapy[10], and protein expression[11] have also been reported.

There are a plethora of prognostic factor studies and prediction tools for prostate cancer risk[12] and prognosis[13] in the published literature. The vast majority are not externally calibrated or validated, and very few are established for use in clinical practice[12]. Initiatives such as the MRC PROGnosis RESearch Strategy Partnership (PROGRESS) partnership highlight the importance of high quality prognostic research to help inform clinical practice[14], and outline methodologically rigorous approaches to achieve this aim[15–17]. Developing clinically useful risk-prediction rules

For peer review only

starts with identifying potentially important prognostic factors which could be incorporated into a prediction model. The aim of the current study is to confirm the association of previously reported prognostic factors with future progression of localised prostate cancer using primary care data and identify new potential prognostic factors for further assessment in prognostic model development and validation.

For peer review only

## Materials and Methods

The protocol for this study has been published previously in *BMJ Open*[18]. In summary, we undertook a retrospective cohort study using a longitudinal dataset of prospectively collected electronic primary care medical records from the Clinical Practice Research Datalink (CPRD)[19]. This dataset was linked with cancer registry data from the National Cancer Research and Analysis Service (NCRAS)[20] and mortality data from the Office for National Statistics (ONS)[21]. Men were included if they had a diagnosis of prostate cancer entered into their medical record during the 20-year study period (01/01/1987 – 31/12/2016). Localised prostate cancer was defined as T1-2/N0/M0 based on staging data entered into the NCRAS registry, which is determined from a combination of clinical, pathological and radiological data[22].

Potentially relevant clinical, biochemical and pharmacological factors measured in CPRD were identified from a review of the existing published literature (See *BMJ Open* protocol paper[18] for more information about the prognostic factors assessed). The primary outcome of the study was prostate cancer mortality. Secondary outcomes were all-cause mortality and commencing systemic prostate cancer therapy (a measurable proxy for progression and metastatic spread of prostate cancer). Surgery, radiotherapy and brachytherapy were classified as localised therapy, with chemotherapy, hormone treatments (primary or neo adjuvant), and immunotherapy considered systemic therapy. In our published protocol[18], up-staging after diagnosis was proposed as a secondary outcome indicating spread of disease; however, this was not used in the final analysis as repeat staging was rarely recorded in the cancer registry (see Table 2).

*Statistical analysis*

Descriptive statistics were used to summarise the basic demographic details of the men, and the prevalence of the pre-selected putative prognostic factors. Cox proportional hazards regression was used to estimate crude and mutually adjusted hazard ratios (with 95% confidence intervals) for prostate cancer specific and all-cause mortality according to the prognostic factors, controlling for variables currently used in clinical practice (age, PSA level, Gleason score). Regression analyses of continuous prognostic factors were standardised using hazard ratios per change in one standard deviation. A Proportional Hazards test was performed to confirm modelling met

For peer review only

regression assumptions. The analysis was also stratified by stage at diagnosis (T1/2N0M0 vs T3+ and/or N1 and/or M1). Sensitivity analysis was performed, assuming all men in the overall sample with unknown tumour location had localised disease. In order to achieve 95% power and detect a difference of 0.1 in prostate cancer mortality for a binary risk factor using an alpha of 0.05, a sample of at least 6,046 men with prostate cancer would be required, assuming that 10% die over a median 10-year follow-up.

*Patient and public involvement*

No patients were involved in the development or design of this study.

*Ethical approval*

This study received ethical approval through the MHRA ISAC process (reference 17_041). The funder had no role in the planning or undertaking of this study, or the preparation of this manuscript.

For peer review only

**Results**

54,500 men within CPRD had a diagnosis of prostate cancer entered into their primary care medical record during the study period. Tumour-Node-Metastases (TNM) staging data from the linked cancer registry were available for 7,646 (14.03%) of the sample population and treatment data were available for 22,766 (41.77%) men. Missing TNM staging data from the cancer registry was lower for men diagnosed in more recent years: there were no TNM stage data for men diagnosed before 1993, rising to 37.2% with TNM stage data (1,064/2,836) in 2015. This is consistent with a recent validation study of the NCRAS prostate cancer registry that showed low levels of completeness of TNM stage and Gleason score data prior to 2010[23]. Using the available staging and treatment data, 10,901 (20%) men were identified as having localised prostate cancer at the time of diagnosis and were included in the final cohort for analysis, with a mean follow-up of 14.12 (+/-6.36) years. Levels of missing data for selected prognostic factors within CPRD varied (see Tables 3 and 4). Baseline participant data are shown in Table 1.

1,250 men with localised disease died of prostate cancer over the course of follow-up, giving a prostate cancer mortality rate of 8.1 per 1,000 person-years. The total number of deaths for included men was 3,250 (21.11 deaths per 1,000 person-years). 2,331 (21.38%) men with localised disease received systemic therapy in the follow-up period after diagnosis. For over 90% of the men it was unknown whether they were re-investigated for cancer staging after diagnosis or not (see Table 2).

Raised acute phase reactants (C-Reactive Protein [CRP] [adjusted HR per SD 1.35 95% CI 1.02, 1.77]), ferritin (adjusted HR per SD 2.03 95% CI 1.21, 3.39) and random glucose (adjusted HR per SD 1.27 95% CI 1.06, 1.54) were associated with prostate cancer mortality. Anaemia (adjusted HR per SD 0.72 95% CI 0.59, 0.88) and low albumin (adjusted HR per SD 0.81 95% CI 0.67, 0.97) were also associated with this outcome. No medications assessed were associated with prostate cancer mortality. Current and ex-smokers (adjusted HR 1.47 95% CI 1.05, 2.05), and patients with a history of ischaemic heart disease (adjusted HR 1.79 95% CI 1.20, 2.66) had a higher risk of prostate cancer mortality over the study period.

For peer review only

Raised CRP, anaemia, and low albumin were biochemical factors associated with all-cause mortality; with anaemia and low albumin also being associated with commencing systemic therapy. A number of other factors were also associated with all-cause mortality, including age, raised PSA, smoking and smoking-related disease, cardiovascular diseases, as well as current use of aspirin or beta blockers. Smoking and beta blockers were also associated with increased risk of systemic therapy, as were vitamin D supplements. Benign prostatic hyperplasia and alpha blocker prescription were associated with a reduced risk of commencing systemic therapy (See Tables 3 & 4 for adjusted analysis results, and Supplementary Tables S1 & S2 for unadjusted results).

Sensitivity analysis including all participants with unknown tumour location showed a relationship between a history of stroke and all-cause mortality (adjusted HR 1.47 95% CI 1.12, 1.93 p = 0.006). The relationship between aspirin and prostate cancer mortality altered to very weak evidence for association (adjusted HR 1.55 95% CI 0.79, 3.02 p = 0.2). For all other factors measured and for all three outcomes in the analysis, the direction of relationship did not change and the magnitude of relationship stayed relatively stable (see Supplementary Tables S3-6).

For peer review only

**Discussion**

This retrospective cohort study utilised primary care medical records data for men with localised prostate cancer from CPRD to confirm prognostic factors associated with prostate cancer progression. Well-known factors already incorporated into clinical guidelines, such as age and PSA, were confirmed as being individual prognostic factors. In addition, further clinical (history of smoking or ischaemic heart disease) and biochemical (anaemia or high ferritin) factors were found to be strongly associated with prostate cancer mortality. Anaemia, low albumin, raised PSA, history of ischaemic heart disease, and smoking were also strongly associated with all-cause mortality, as were peripheral vascular disease, COPD, and beta blocker use. Smoking history was strongly associated with future systemic therapy, as were recent prescriptions of alpha blockers, or vitamin D supplements.

This analysis confirms the prognostic associations of some factors in prostate cancer progression. Smoking has also been found to be a risk factor for prostate cancer progression and mortality in cohort studies[24] and systematic reviews[8]. Low albumin was associated with prostate cancer mortality in the AMORIS cohort[25] and, along with anaemia[26], is a more widely accepted predictor of poor cancer outcomes[27]. The published literature around the prognostic effect of beta blockers for prostate cancer patients has been more mixed[28], with this study lending weight to the evidence of increased mortality in cancer patients. BMI was not shown to be associated with prostate cancer and overall mortality in this study. Whilst some observational studies of prostate cancer have suggested an association may exist[8,29,30], reviews of trial data have demonstrated higher BMI may actually improve the prognosis for men with cancer[31].

This study attempted to confirm prognostic factors in a primary care dataset that could be used in a model to predict prostate cancer progression at the time of diagnosis, prior to any treatment being initiated. This approach could allow the identified prognostic factors to be used to develop a new prognostic tool to inform treatment decisions between a patient and their treating team. There are already examples of similar prognostic tools available for use, including Predict Prostate (https://prostate.predict.nhs.uk/). However, these tools have only been developed using secondary care data[32], which may not capture all important prognostic factors or have

For peer review only - http://bmjopen.bmj.com/site/about/guidelines.xhtml

equivalent length of follow-up of patients in their development or calibration cohorts. In the context of on-going challenges with prognostication for men with localised prostate cancer, and the increasing numbers of men being diagnosed every year, getting the most accurate information to inform treatment discussions between patients and their treating physicians is vital.

*Strengths and limitations*

This study has a number of unique features. This is the first study that the authors are aware of to utilise a primary care dataset to identify and confirm prognostic factors associated with prostate cancer progression. CPRD contains all data held in the primary care records of millions of UK patients, allowing the inclusion of a range of potentially important prognostic factors. Using a primary care dataset from the NHS also provided long-term data for included patients, with a mean follow-up of over 14 years. Prolonged follow-up for men with prostate cancer is important as many patients can live for years before their cancer progresses. The lack of high quality prognostic research discussed in the introduction is not limited to prostate cancer, with many other prognostic factor studies being conducted in similarly flawed ways[33–35]. This study sought to take a confirmatory approach to postulated prognostic factors in prostate cancer in a rigorous manner, following the methodological recommendations of the REMARK guidelines[36] and the PROGRESS partnership[14–17].

There are some limitations of this study that need to be considered. Previous research has shown that the prostate cancer registry in England has strong case completeness, but significant missing TNM stage and Gleason score data up until recent years[23]. Data completeness and quality within NCRAS continues to improve, and there is no equivalent UK cancer registry dataset with more complete data available at this present time[22]. This level of missing data meant it was unknown whether the majority of potentially included men had localised disease or not. Even so, the study was still powered to answer the research question, and sensitivity analyses showed minimal changes to almost all relationships between the prognostic factors of interest and the study outcomes. This study uses a retrospective design interrogating electronic primary care records. It relies on accurate coding from GPs[37], and there was significant missing data for some prognostic factors.

For peer review only - http://bmjopen.bmj.com/site/about/guidelines.xhtml

This study took a confirmatory approach to identify which prognostic factors for prostate cancer progression may be relevant, and some new prognostic factors not currently recommended for use in clinical practice were identified. These prognostic factors could be used to generate a more robust clinical risk prediction tool to guide treatment decision-making. Developing an accurate prediction tool for prostate cancer progression, not just mortality, could be more useful for informing management discussions between patients and clinicians.

For peer review only

**Ethical approval**

This study received ethical approval from the Independent Scientific Advisory Committee (ISAC) of the Medicines and Healthcare products Regulatory Authority (MHRA) – Protocol reference 17_041. It was conducted in accordance with the Declaration of Helsinki.

**Funding statement**

This work was supported from an Academic Clinical Fellowship in Primary Care for SWDM, funded by the National Institute for Health Research and Health Education England. The views expressed in this publication are those of the author(s) and not necessarily those of the NHS, the National Institute for Health Research, Health Education England or the Department of Health. RMM was supported by a CRUK programme grant, the Integrative Cancer Epidemiology Programme (C18281/A19169). MTM was supported by the NIHR Biomedical Research Centre at University Hospitals Bristol NHS Foundation Trust and the University of Bristol.

**Author statement**

SWDM conceived and designed the work that has led to this submission. He acquired the data and performed the analysis. He drafted the manuscript and approves the final version. He agrees to be accountable for all aspects of the work. As corresponding author, he also confirms he has full access to the data in the study and has taken final responsibility for the decision to submit for publication.

SMI played an important role in the data analysis and interpretation of the results. She revised the manuscript and approved the final version. She agrees to be accountable for all aspects of the work.

MTM helped design the work that has led to this submission, and supported interpretation of the results. She also provided study supervision to SWDM. She has revised the manuscript and approved the final version. She agrees to be accountable for all aspects of the work.

RMM helped to conceive and design the work that has led to this submission. He also provided study supervision to SWDM He has revised the manuscript and approved the final version. He agrees to be accountable for all aspects of the work.

For peer review only

**Conflict of interest disclosure statement** – The authors declare no potential conflicts of interest

**Data statement**

This study analysed a CPRD dataset, with linked NCRAS and ONS data. Permission was not sought to share the dataset publicly.

For peer review only

## References


1 Collin SM, Martin RM, Metcalfe C, *et al.* Prostate-cancer mortality in the USA and UK in 1975-2004: an ecological study. *Lancet Oncol* 2008;**9**:445–52. doi:10.1016/S1470-2045(08)70104-9

2 Ferlay J, Soerjomataram I, Ervik M, *et al.* GLOBOCAN 2012 v1.0, Cancer Incidence and Mortality Worldwide: IARC CancerBase No. 11 [Internet]. Int. Agency Res. Cancer. 2013.http://globocan.iarc.fr (accessed 24 May 2018).

3 Sandhu GS, Andriole GL. Overdiagnosis of prostate cancer. *J Natl Cancer Inst - Monogr* 2012;**2012**:146–51. doi:10.1093/jncimonographs/lgs031

4 Donovan JL, Hamdy FC, Lane JA, *et al.* Patient-Reported Outcomes after Monitoring, Surgery, or Radiotherapy for Prostate Cancer. *N Engl J Med* 2016;**375**:1425–37. doi:10.1056/NEJMoa1606221

5 Hamdy FC, Donovan JL, Lane JA, *et al.* 10-Year Outcomes after Monitoring, Surgery, or Radiotherapy for Localized Prostate Cancer. *N Engl J Med* 2016;:NEJMoa1606220-10.http://www.nejm.org/doi/10.1056/NEJMoa1606220

6 Therasse P, Arbuck SG, Eisenhauer EA, *et al.* New guidelines to evaluate the response to treatment in solid tumors. European Organization for Research and Treatment of Cancer, National Cancer Institute of the United States, National Cancer Institute of Canada. JNCI J. Natl. Cancer Inst. 2000;**92**:205–16.http://jnci.oxfordjournals.org/cgi/doi/10.1093/jnci/92.3.205

7 Eisenhauer EA, Therasse P, Bogaerts J, *et al.* New response evaluation criteria in solid tumours: Revised RECIST guideline (version 1.1). 2009;**45**:228–47.http://dx.doi.org/10.1016/j.ejca.2008.10.026

8 Peisch SF, Blarigan EL Van, Chan JM, *et al.* Prostate cancer progression and mortality: a review of diet and lifestyle factors. *World J Urol* 2017;**35**:867–74. doi:10.1007/s00345-016-1914-3.Prostate

9 Bianco-Miotto T, Chiam K, Buchanan G, *et al.* Global levels of specific histone modifications and an epigenetic gene signature predict prostate cancer progression and development. *Cancer Epidemiol Biomarkers Prev* 2010;**19**:2611–22. doi:10.1158/1055-9965.EPI-10-0555

10 Fleshner N, Lucia MS, Melich K, *et al.* Effect of dutasteride on prostate cancer progression


For peer review only

For peer review only - http://bmjopen.bmj.com/site/about/guidelines.xhtml

and cancer diagnosis on rebiopsy in the REDEEM active surveillance study. *J Clin Oncol* 2011;**29**:no pagination. doi:10.1200/jco.2011.29.7

11 Cullen J, Young D, Chen Y, *et al.* Predicting Prostate Cancer Progression as a Function of ETS-related Gene Status, Race, and Obesity in a Longitudinal Patient Cohort. *Eur Urol Focus* 2017;**1**:1–7. doi:10.1016/j.euf.2017.02.016

12 Louie KS, Seigneurin A, Cathcart P, *et al.* Do prostate cancer risk models improve the predictive accuracy of PSA screening? A meta-analysis. *Ann Oncol* 2015;**26**:848–64. doi:10.1093/annonc/mdu525

13 Shariat SF, Kattan MW, Vickers AJ, *et al.* Critical review of prostate cancer predictive tools. *Futur Oncol* 2009;**5**:1555–84.http://www.futuremedicine.com/doi/10.2217/fon.09.121

14 Hemingway H, Croft P, Perel P, *et al.* Prognosis research strategy (PROGRESS) 1: A framework for researching clinical outcomes. *BMJ* 2013;**346**:e5595–e5595.http://www.bmj.com/cgi/doi/10.1136/bmj.e5595

15 Riley RD, Hayden JA, Steyerberg EW, *et al.* Prognosis Research Strategy (PROGRESS) 2: Prognostic Factor Research. *PLoS Med* 2013;**10**:e1001380-9.http://dx.plos.org/10.1371/journal.pmed.1001380

16 Steyerberg EW, Moons KGM, van der Windt DA, *et al.* Prognosis Research Strategy (PROGRESS) 3: Prognostic Model Research. *PLoS Med* 2013;**10**:e1001381-9.http://dx.plos.org/10.1371/journal.pmed.1001381

17 Hingorani AD, Windt DA v d, Riley RD, *et al.* Prognosis research strategy (PROGRESS) 4: Stratified medicine research. *BMJ* 2013;**346**:e5793–e5793.http://www.bmj.com/cgi/doi/10.1136/bmj.e5793

18 Merriel SWD, May MT, Martin RM. Predicting prostate cancer progression: protocol for a retrospective cohort study to identify prognostic factors for prostate cancer outcomes using routine primary care data. *BMJ Open* 2018;**8**:e019409. doi:10.1136/ bmjopen-2017-019409

19 CPRD. Welcome to the Clinical Practice Research Datalink. https://www.cprd.com/home/

20 NCRAS. National Cancer Research and Analysis Service. papers3://publication/uuid/CBEF1C52-AEBE-4B92-A099-2BC799944207

21 ONS. Deaths Registered in England and Wales (Series DR), 2016. London: 2017. http://www.ons.gov.uk/ons/rel/vsob1/mortality-statistics--deaths-registered-in-england-


For peer review only

and-wales--series-dr-/2014/stb-mortality-stats-2014.html

22 Henson KE, Elliss-Brookes L, Coupland VH, *et al.* Data Resource Profile: National Cancer Registration Dataset in England. *Int J Epidemiol* 2020;**49**:16-16h. doi:10.1093/ije/dyz076

23 Merriel SWD, Turner EL, Walsh E, *et al.* Cross-sectional study evaluating data quality of the National Cancer Registration and Analysis Service (NCRAS) prostate cancer registry data using the Cluster randomised trial of PSA testing for Prostate cancer (CAP). *BMJ Open* 2017;**7**:e015994. doi:10.1136/bmjopen-2017-015994

24 Rohrmann S, Genkinger JM, Burke A, *et al.* Smoking and Risk of Fatal Prostate Cancer in a Prospective U.S. Study. *Urology* 2007;**69**:721–5.http://linkinghub.elsevier.com/retrieve/pii/S0090429506026446

25 Arthur R, Williams R, Garmo H, *et al.* Serum inflammatory markers in relation to prostate cancer severity and death in the Swedish AMORIS study. *Int J Cancer* 2018;**142**:2254–62. doi:10.1002/ijc.31256

26 Van Belle SJP. What is the value of hemoglobin as a prognostic and predictive factor in cancer? *Eur J Cancer, Suppl* 2004;**2**:11–9. doi:10.1016/S1359-6349(03)00103-4

27 Gupta D, Lis CG. Pretreatment serum albumin as a predictor of cancer survival: A systematic review of the epidemiological literature. *Nutr J* 2010;**9**:1–16. doi:10.1186/1475-2891-9-69

28 Assayag J, Pollak MN, Azoulay L. Post-diagnostic use of beta-blockers and the risk of death in patients with prostate cancer. *Eur J Cancer* 2014;**50**:2838–45.http://dx.doi.org/10.1016/j.ejca.2014.08.006

29 Cantarutti A, Bonn SE, Adami H-O, *et al.* Body mass index and mortality in men with prostate cancer. *Prostate* 2015;**75**:1129–36. doi:10.1002/pros.23001

30 Haque R, Van Den Eeden SK, Wallner LP, *et al.* Association of body mass index and prostate cancer mortality. *Obes Res Clin Pract* 2014;**8**:e374-81. doi:10.1016/j.orcp.2013.06.002

31 Greenlee H, Unger JM, LeBlanc M, *et al.* Association between body mass index and cancer survival in a pooled analysis of 22 clinical trials. *Cancer Epidemiol Biomarkers Prev* 2017;**26**:21–9. doi:10.1158/1055-9965.EPI-15-1336

32 Gnanapragasam VJ, Lophatananon A, Wright KA, *et al.* Improving Clinical Risk Stratification at Diagnosis in Primary Prostate Cancer: A Prognostic Modelling Study. *PLoS Med* 2016;**13**:1–18. doi:10.1371/journal.pmed.1002063

For peer review only

33 Kyzas PA, Denaxa-Kyza D, Ioannidis JPA. Almost all articles on cancer prognostic markers report statistically significant results. *Eur J Cancer* 2007;**43**:2559–79. doi:10.1016/j.ejca.2007.08.030

34 Kyzas PA, Loizou KT, Ioannidis JPA. Selective reporting biases in cancer prognostic factor studies. *J Natl Cancer Inst* 2005;**97**:1043–55. doi:10.1093/jnci/dji184

35 Hemingway H, Riley RD, Altman DG. Ten steps towards improving prognosis research. *BMJ* 2010;**340**:410–4. doi:10.1136/bmj.b4184

36 McShane LM, Altman DG, Sauerbrei W, *et al.* Reporting Recommendations for Tumor Marker Prognostic Studies (REMARK). *JNCI J Natl Cancer Inst* 2005;**97**:1180–4.https://academic.oup.com/jnci/article-lookup/doi/10.1093/jnci/dji237

37 Reeves D, Springate DA, Ashcroft DM, *et al.* Can analyses of electronic patient records be independently and externally validated? The effect of statins on the mortality of patients with ischaemic heart disease: a cohort study with nest case-control analysis. *BMJ Open* 2014;**4**:1–11.http://dx.doi.org/10.1136/bmjopen-2014-004952

For peer review only

| | **Localised**<br>**n = 10,901** | **Missing data** |
|---|---|---|
| Mean (SD) | | |
| Age (yrs) | 74.38 (+/- 9.03) | 0% |
| BMI (kg/m$^2$) | 27.43 (+/- 4.48) | 5.64% |
| Follow-up (yrs) | 14.12 (+/- 6.36) | 0% |
| Median (IQR) | | |
| PSA (ng/mL) | 8.4 (5.55, 14.6) | 30.66% |
| n (%) | | |
| Gleason score | | |
| 6 | 3,655 (33.53%) | 33.23% |
| 7+ | 4,420 (40.55%) | |
| Family history of prostate cancer | 70 (0.64%) | 55.11% |
| Ethnicity | | |
| White | 7,361 (67.53%) | 29.79% |
| Mixed | 21 (0.19%) | |
| Asian | 75 (0.69%) | |
| Black | 156 (1.43%) | |
| Other | 41 (0.38%) | |

Table 1 – Baseline participant data

For peer review only

| | | Prostate cancer mortality | All-cause mortality | Systemic therapy | Upstaging[a] |
|---|---|---|---|---|---|
| Included | Localised (T1/2 N0 M0) n = 10,901 | 1,250 (11.47%) | 3,450 (31.65%) | 2,331 (21.38%) | 45 (0.41%) |
| Excluded | Invasive (T3+ / N1 / M1) n = 12,318 | 3,894 (31.61%) | 6,916 (56.15%) | 10,881 (88.33%) | 28 (0.23%) |
| | Unknown n = 31,281 | 1,540 (4.92%) | 5,420 (17.33%) | 31,954 (58.63%) | 19 (0.06%) |

Table 2 – Primary and Secondary outcomes for included and excluded participants

[a] Repeat staging data missing for 50,119 (91.96%) of sample

For peer review only

| n = 10,901 | | | Prostate cancer mortality | | | All-cause mortality | | | Systemic therapy | | |
|---|---|---|---|---|---|---|---|---|---|---|---|
| **Factor** | **Mean (SD)** | **Missing [n (%)]** | **HR per SD[a]** | **95% CI** | **p** | **HR per SD[a]** | **95% CI** | **p** | **HR per SD[a]** | **95% CI** | **p** |
| Age | 74.39 (9.03) | 0 | 1.70 | 1.40, 2.06 | <0.01 | 1.92 | 1.74, 2.12 | <0.01 | 1 | 0.95, 1.06 | 0.98 |
| BMI | 27.43 (4.48) | 394 (3.61%) | 1.05 | 0.90, 1.08 | 0.52 | 0.97 | 0.90, 1.05 | 0.51 | 1.04 | 0.99, 1.09 | 0.10 |
| Triglycerides | 1.45 (0.80) | 3,856 (35.37%) | 0.83 | 0.64, 1.08 | 0.16 | 1.00[b] | 0.90, 1.13 | 0.93 | 1.03 | 0.97, 1.09 | 0.37 |
| HDL cholesterol | 1.35 (0.43) | 3,954 (36.27%) | 1.05 | 0.89, 1.23 | 0.56 | 1.01 [b] | 0.91, 1.12 | 0.86 | 1.01 | 0.95, 1.07 | 0.75 |
| LDL cholesterol | 2.95 (0.99) | 4,698 (43.10%) | 0.86 | 0.69, 1.07 | 0.18 | 0.92 [b] | 0.82, 1.02 | 0.12 | 0.99 | 0.94, 1.05 | 0.86 |
| Hb | 144.28 (14.35) | 2,696 (24.73%) | 0.72 | 0.59, 0.88 | <0.01 | 0.74 | 0.67, 0.82 | <0.01 | 0.92 | 0.86, 0.98 | 0.01 |
| Albumin | 41.83 (3.94) | 2,954 (27.10%) | 0.81 | 0.67, 0.97 | 0.02 | 0.83 | 0.76, 0.91 | <0.01 | 0.94 | 0.89, 0.99 | 0.04 |
| Random glucose | 5.70 (2.11) | 4,525 (41.51%) | 1.27 | 1.06, 1.54 | 0.01 | 1.12 | 0.99, 1.25 | 0.06 | 1.02[b] | 0.95, 1.09 | 0.66 |
| | **Median (IQR)** | **Missing [n (%)]** | | | | | | | | | |
| PSA | 8.4 (5.55, 14.60) | 2,352 (21.58%) | 1.71 | 1.32, 2.23 | <0.01 | 1.46 | 1.19, 1.78 | <0.01 | 1.34 | 1.06, 1.68 | 0.01 |
| CRP | 3.9 (2, 8) | 8,061 (73.95%) | 1.35 [b] | 1.02, 1.77 | 0.03 | 1.23 [b] | 1.05, 1.45 | 0.01 | 1.07 | 0.95, 1.20 | 0.24 |
| Ferritin | 108.6 (47, 196) | 9,495 (87.10%) | 2.03 | 1.21, 3.39 | <0.01 | 0.98 [b] | 0.60, 1.59 | 0.93 | 1.05 | 0.85, 1.31 | 0.64 |

Table 3 – Prognostic factors for men with localised disease associated with outcomes

[a] Adjusted for age, PSA, Gleason score, TNM stage

[b] Proportional Hazards assumption test not met

HR – Hazard Ratio; SD – Standard Deviation; BMI – Body Mass Index; PSA – Prostate Specific Antigen; HDL – High Density Lipoprotein; LDL – Low Density Lipoprotein; HbA1c – Haemoglobin A1c; CRP – C-Reactive Protein; Hb – Haemoglobin

For peer review only

For peer review only - http://bmjopen.bmj.com/site/about/guidelines.xhtml

| n = 10,901 | | | Prostate cancer mortality | | | All-cause mortality | | | Systemic therapy | | |
|---|---|---|---|---|---|---|---|---|---|---|---|
| **Factor** | **n (%)** | **Missing [n(%)]** | **HR[a]** | **95% CI** | **p** | **HR[a]** | **95% CI** | **p** | **HR[a]** | **95% CI** | **p** |
| Smoker (current/ ex-) | 5,112 (46.89%) | 777 (7.13%) | 1.47 | 1.05, 2.05 | 0.02 | 1.66 | 1.39, 1.98 | <0.01 | 1.21 | 1.09, 1.33 | <0.01 |
| Excess alcohol | 1,829 (16.78%) | 4,370 (40.09%) | 0.61 | 0.36, 1.04 | 0.07 | 0.91 [b] | 0.71, 1.18 | 0.47 | 0.99 | 0.87, 1.13 | 0.88 |
| BPH | 1,169 (10.72%) | 3,526 (32.35%) | 0.64 | 0.36, 1.11 | 0.11 | 0.81 | 0.62, 1.05 | 0.11 | 0.76 | 0.65, 0.90 | <0.01 |
| COPD | 862 (7.91%) | 3,583 (32.87%) | 0.86 | 0.47, 1.57 | 0.63 | 1.64 | 1.29, 2.09 | <0.01 | 1.18 | 0.99, 1.41 | 0.06 |
| CVA | 553 (5.07%) | 3,584 (32.88%) | 0.90 | 0.42, 1,94 | 0.79 | 1.19 | 0.85, 1.68 | 0.30 | 0.92 | 0.72, 1.17 | 0.49 |
| IHD | 1,548 (14.20%) | 3,405 (31.24%) | 1.79 | 1.20, 2.66 | <0.01 | 1.25 | 1.02, 1.55 | 0.04 | 1.01 | 0.87, 1.18 | 0.86 |
| PVD | 202 (1.85%) | 3,582 (32.86%) | 2.24 | 0.98, 5.12 | 0.06 | 1.91 | 1.24, 2.95 | <0.01 | 1.04 | 0.71, 1.51 | 0.85 |
| T2DM | 1,508 (13.83%) | 3,448 (31.63%) | 0.97 | 0.62, 1.51 | 0.89 | 0.95 | 0.76, 1.19 | 0.68 | 0.99 | 0.86, 1.14 | 0.91 |
| Aspirin | 426 (3.91%) | 16 (0.15%) | 1.88 | 0.96, 3.70 | 0.06 | 1.58 | 1.09, 2.29 | 0.02 | 1.24 | 0.95, 1.60 | 0.11 |
| Metformin | 33 (0.30%) | | | | | 2.74 | 0.88, 8.49 | 0.08 | 1.73 | 0.77, 3.85 | 0.18 |
| Alpha blockers | 305 (2.80%) | | 1.55 | 0.72, 3.35 | 0.26 | 1.15 | 0.76, 1.73 | 0.52 | 0.57 | 0.39, 0.82 | <0.01 |
| Beta blockers | 265 (2.43%) | | 2.03 | 0.89, 4.60 | 0.09 | 1.79 | 1.18, 2.72 | <0.01 | 1.48 | 1.09, 1.99 | 0.01 |
| Statins | 339 (3.11%) | | 1.65 | 0.87, 3.15 | 0.13 | 1.01 | 0.66, 1.53 | 0.97 | 1.06 | 0.84, 1.34 | 0.61 |
| Vitamin D | 465 (4.27%) | | 1.33 | 0.65, 2.71 | 0.44 | 1.13 | 0.78, 1.65 | 0.51 | 1.35 | 1.09, 1.68 | <0.01 |

Table 4 – Prognostic factors for men with localised disease associated with outcomes

[a] Adjusted for age, PSA, Gleason score, TNM stage

[b] Proportional Hazards assumption test not met

HR – Hazard Ratio; BPH – Benign Prostatic Hypertrophy; COPD – Chronic Obstructive Pulmonary Disease; CVA – Cerebrovascular Accident; IHD – Ischaemic Heart Disease; PVD – Peripheral Vascular Disease; T2DM – Type 2 Diabetes Mellitus

For peer review only

| n = 10,901 | | | Prostate cancer mortality | | | All-cause mortality | | | Systemic therapy | | |
|---|---|---|---|---|---|---|---|---|---|---|---|
| **Factor** | **Mean (SD)** | **Missing [n (%)]** | **HR per SD** | **95% CI** | **p** | **HR per SD** | **95% CI** | **p** | **HR per SD** | **95% CI** | **p** |
| Age | 74.39 (9.03) | 0 | 1.66 | 1.56, 1.76 | <0.01 | 1.82 | 1.76, 1.89 | <0.01 | 0.81 | 0.77, 0.84 | <0.01 |
| BMI | 27.43 (4.48) | 394 (3.61%) | 0.96 | 0.91, 1.02 | 0.19 | 0.97 | 0.94, 1.01 | 0.15 | 1.07 | 1.03, 1.12 | <0.01 |
| Triglycerides | 1.45 (0.80) | 3,856 (35.37%) | 0.93 | 0.84, 1.02 | 0.12 | 0.97 | 0.92, 1.02 | 0.28 | 0.99 | 0.95, 1.04 | 0.82 |
| HDL cholesterol | 1.35 (0.43) | 3,954 (36.27%) | 0.98 | 0.88, 1.09 | 0.66 | 0.98 | 0.92, 1.04 | 0.49 | 1.03 | 0.98, 1.08 | 0.23 |
| LDL cholesterol | 2.95 (0.99) | 4,698 (43.10%) | 0.90 | 0.81, 0.99 | 0.03 | 0.87 | 0.82, 0.92 | <0.01 | 0.95 | 0.91, 0.99 | 0.03 |
| Hb | 144.28 (14.35) | 2,696 (24.73%) | 0.55 | 0.51, 0.58 | <0.01 | 0.62 | 0.59, 0.64 | <0.01 | 0.95 | 0.89, 0.99 | 0.03 |
| Albumin | 41.83 (3.94) | 2,954 (27.10%) | 0.71 | 0.67, 0.74 | <0.01 | 0.74 | 0.71, 0.77 | <0.01 | 0.96 | 0.91, 1.00 | 0.06 |
| Random glucose | 5.70 (2.11) | 4,525 (41.51%) | 1.18 | 1.10, 1.27 | <0.01 | 1.14 | 1.09, 1.20 | <0.01 | 1.02 | 0.97, 1.07 | 0.48 |
| | **Median (IQR)** | **Missing [n (%)]** | | | | | | | | | |
| PSA | 8.4 (5.55, 14.60) | 2,352 (21.58%) | 1.45 | 1.39, 1.51 | <0.01 | 1.36 | 1.31, 1.41 | <0.01 | 1.01 | 0.86, 1.18 | 0.90 |
| CRP | 3.9 (2, 8) | 8,061 (73.95%) | 1.27 | 1.19, 1.36 | <0.01 | 1.22 | 1.16, 1.28 | <0.01 | 1.01 | 0.92, 1.10 | 0.90 |
| Ferritin | 108.6 (47, 196) | 9,495 (87.10%) | 1.48 | 1.25, 1.75 | <0.01 | 1.13 | 0.97, 1.31 | 0.11 | 1.02 | 0.89, 1.16 | 0.79 |

Table S1 – Unadjusted HRs for prognostic factors for men with localised disease associated with outcomes

HR – Hazard Ratio; SD – Standard Deviation; BMI – Body Mass Index; PSA – Prostate Specific Antigen; HDL – High Density Lipoprotein; LDL – Low Density Lipoprotein; HbA1c – Haemoglobin A1c; CRP – C-Reactive Protein; Hb – Haemoglobin

For peer review only

| n = 10,901 | | | Prostate cancer mortality | | | All-cause mortality | | | Systemic therapy | | |
|---|---|---|---|---|---|---|---|---|---|---|---|
| **Factor** | **n (%)** | **Missing [n(%)]** | **HR** | **95% CI** | **p** | **HR** | **95% CI** | **p** | **HR** | **95% CI** | **p** |
| Smoker (current/ ex-) | 5,112 (46.89%) | 777 (7.13%) | 1.24 | 1.09, 1.40 | <0.01 | 1.36 | 1.26, 1.46 | <0.01 | 1.20 | 1.10, 1.30 | <0.01 |
| Excess alcohol | 1,829 (16.78%) | 4,370 (40.09%) | 0.75 | 0.62, 0.91 | <0.01 | 0.67 | 0.60, 0.74 | <0.01 | 0.92 | 0.80, 1.06 | 0.26 |
| BPH | 1,169 (10.72%) | 3,526 (32.35%) | 0.61 | 0.49, 0.76 | <0.01 | 0.79 | 0.70, 0.88 | <0.01 | 0.70 | 0.61, 0.80 | <0.01 |
| COPD | 862 (7.91%) | 3,583 (32.87%) | 0.95 | 0.77, 1.18 | 0.67 | 1.41 | 1.26, 1.58 | <0.01 | 1.08 | 0.94, 1.25 | 0.26 |
| CVA | 553 (5.07%) | 3,584 (32.88%) | 1.48 | 1.18, 1.85 | <0.01 | 1.89 | 1.67, 2.13 | <0.01 | 0.78 | 0.64, 0.96 | 0.02 |
| IHD | 1,548 (14.20%) | 3,405 (31.24%) | 1.62 | 1.39, 1.88 | <0.01 | 1.79 | 1.64, 1.95 | <0.01 | 0.89 | 0.79, 1.00 | 0.05 |
| PVD | 202 (1.85%) | 3,582 (32.86%) | 1.80 | 1.31, 2.49 | <0.01 | 1.87 | 1.55, 2.25 | <0.01 | 0.84 | 0.62, 1.14 | 0.26 |
| T2DM | 1,508 (13.83%) | 3,448 (31.63%) | 1.13 | 0.96, 1.33 | 0.16 | 1.03 | 0.94, 1.14 | 0.49 | 0.93 | 0.83, 1.04 | 0.20 |
| Aspirin | 426 (3.91%) | 16 (0.15%) | 2.33 | 1.88, 2.90 | <0.01 | 2.18 | 1.90, 2.49 | <0.01 | 1.24 | 0.99, 1.54 | 0.06 |
| Metformin | 33 (0.30%) | | 1.99 | 0.90, 4.46 | 0.09 | 2.63 | 1.71, 4.04 | <0.01 | 2.59 | 1.47, 4.57 | <0.01 |
| Alpha blockers | 305 (2.80%) | | 1.24 | 0.92, 1.67 | 0.16 | 1.17 | 0.97, 1.41 | 0.09 | 0.51 | 0.37, 0.71 | <0.01 |
| Beta blockers | 265 (2.43%) | | 2.15 | 1.65, 2.81 | <0.01 | 1.76 | 1.48, 2.10 | <0.01 | 1.11 | 0.85, 1.46 | 0.43 |
| Statins | 339 (3.11%) | | 1.34 | 1.01, 1.77 | 0.04 | 0.93 | 0.76, 1.13 | 0.45 | 1.42 | 1.16, 1.73 | <0.01 |
| Vitamin D | 465 (4.27%) | | 1.63 | 1.29, 2.05 | <0.01 | 1.24 | 1.06, 1.46 | <0.01 | 1.52 | 1.27, 1.82 | <0.01 |

Table S2 – Unadjusted HRs for prognostic factors for men with localised disease associated with outcomes

HR – Hazard Ratio; BPH – Benign Prostatic Hypertrophy; COPD – Chronic Obstructive Pulmonary Disease; CVA – Cerebrovascular Accident; IHD – Ischaemic Heart Disease; PVD – Peripheral Vascular Disease; T2DM – Type 2 Diabetes Mellitus

For peer review only

| **n = 42, 182** | | | **Prostate cancer mortality** | | | **All-cause mortality** | | | **Systemic therapy** | | |
|---|---|---|---|---|---|---|---|---|---|---|---|
| **Factor** | **Mean (SD)** | **Missing [n (%)]** | **HR per SD** | **95% CI** | **p** | **HR per SD** | **95% CI** | **p** | **HR per SD** | **95% CI** | **p** |
| Age | 76.70 (9.42) | 0 | 1.55 | 1.49, 1.62 | <0.01 | 1.78 | 1.74, 1.82 | <0.01 | 0.67 | 0.65, 0.70 | <0.01 |
| BMI | 27.37 (4.49) | 394 (3.61%) | 0.96 | 0.92, 0.99 | 0.05 | 0.96 | 0.94, 0.98 | <0.01 | 1.06 | 1.02, 1.10 | <0.01 |
| Triglycerides | 1.48 (0.82) | 3,856 (35.37%) | 0.92 | 0.87, 0.99 | 0.02 | 0.95 | 0.91, 0.98 | <0.01 | 0.96 | 0.91, 1.00 | 0.06 |
| HDL cholesterol | 1.34 (0.53) | 3,954 (36.27%) | 1 | 0.94, 1.07 | 0.60 | 1 | 0.97, 1.04 | 0.80 | 1.03 | 0.99, 1.06 | 0.12 |
| LDL cholesterol | 2.84 (0.99) | 4,698 (43.10%) | 0.93 | 0.86, 0.99 | 0.03 | 0.89 | 0.86, 0.93 | <0.01 | 1.05 | 1, 1.10 | 0.03 |
| Hb | 141.81 (16.58) | 2,696 (24.73%) | 0.61 | 0.58, 0.63 | <0.01 | 0.66 | 0.64, 0.67 | <0.01 | 1.07 | 1.02, 1.13 | <0.01 |
| Albumin | 41.41 (4.31) | 2,954 (27.10%) | 1.71 | 0.99, 2.96 | 0.06 | 1.33 | 0.91, 1.94 | 0.15 | 0.75 | 0.46, 1.21 | 0.24 |
| Random glucose | 5.92 (2.11) | 4,525 (41.51%) | 1.06 | 1.01, 1.10 | 0.01 | 1.07 | 1.04, 1.09 | <0.01 | 0.92 | 0.87, 0.98 | 0.01 |
| | **Median (IQR)** | **Missing [n (%)]** | | | | | | | | | |
| PSA | 10.4 (6.11, 24.6) | 2,352 (21.58%) | 1.28 | 0.96, 1.71 | 0.10 | 0.84 | 0.73, 0.98 | 0.02 | 2.37 | 1.88, 2.99 | <0.01 |
| CRP | 5 (2, 10) | 8,061 (73.95%) | 2.50 | 1.03, 6.08 | 0.04 | 2.55 | 1.47, 4.42 | <0.01 | 1.03 | 0.51, 2.06 | 0.94 |
| Ferritin | 101.5 (45, 197) | 9,495 (87.10%) | 1.10 | 1.06, 1.15 | <0.01 | 1.04 | 0.99, 1.09 | 0.12 | 0.99 | 0.90, 1.09 | .089 |

Table S3 – Unadjusted HRs for prognostic factors for men with localised disease and unknown location associated with outcomes

HR – Hazard Ratio; SD – Standard Deviation; BMI – Body Mass Index; PSA – Prostate Specific Antigen; HDL – High Density Lipoprotein; LDL – Low Density Lipoprotein; HbA1c – Haemoglobin A1c; CRP – C-Reactive Protein; Hb – Haemoglobin

For peer review only

| n = 42, 182 | | | Prostate cancer mortality | | | All-cause mortality | | | Systemic therapy | | |
|---|---|---|---|---|---|---|---|---|---|---|---|
| **Factor** | **n (%)** | **Missing [n(%)]** | **HR** | **95% CI** | **p** | **HR** | **95% CI** | **p** | **HR** | **95% CI** | **p** |
| Smoker (current/ ex-) | 19,215 (45.56%) | 777 (7.13%) | 1.24 | 1.09, 1.40 | <0.01 | 1.36 | 1.26, 1.46 | <0.01 | 1.20 | 1.10, 1.30 | <0.01 |
| Excess alcohol | 5,926 (14.05%) | 4,370 (40.09%) | 0.75 | 0.62, 0.90 | <0.01 | 0.79[b] | 0.71, 0.88 | <0.01 | 1.01 | 0.90, 1.13 | 0.86 |
| BPH | 4,318 (10.24%) | 3,526 (32.35%) | 0.61 | 0.49, 0.76 | <0.01 | 0.79 | 0.70, 0.89 | <0.01 | 0.70 | 0.61, 0.80 | <0.01 |
| COPD | 3,866 (9.17%) | 3,583 (32.87%) | 0.95 | 0.77, 1.19 | 0.66 | 1.41 | 1.26, 1.58 | <0.01 | 1.08 | 0.94, 1.25 | 0.26 |
| CVA | 2,973 (7.05%) | 3,584 (32.88%) | 1.48 | 1.19, 1.85 | <0.01 | 1.89 | 1.67, 2.12 | <0.01 | 0.78 | 0.64, 0.96 | 0.02 |
| IHD | 7,512 (17.81%) | 3,405 (31.24%) | 1.62 | 1.39, 1.88 | <0.01 | 1.79 | 1.64, 1.95 | <0.01 | 0.89 | 0.79, 1.00 | 0.05 |
| PVD | 1,138 (2.70%) | 3,582 (32.86%) | 1.8 | 1.31, 2.49 | <0.01 | 1.89 | 1.55, 2.25 | <0.01 | 0.84 | 0.62, 1.14 | 0.26 |
| T2DM | 6,233 (14.78%) | 3,448 (31.63%) | 1.13 | 0.96, 1.33 | 0.16 | 1.04 | 0.94, 1.14 | 0.49 | 0.93 | 0.83, 1.04 | 0.20 |
| Aspirin | 2,022 (4.79%) | 16 (0.15%) | 2.33 | 1.88, 2.90 | <0.01 | 2.18 | 1.90, 2.49 | <0.01 | 1.24 | 0.99, 1.54 | 0.06 |
| Metformin | 220 (0.52%) | | 1.99 | 0.90, 4.46 | 0.09 | 2.63 | 1.71, 4.04 | <0.01 | 2.59 | 1.47, 4.57 | <0.01 |
| Alpha blockers | 1,025 (2.43%) | | 1.24 | 0.92, 1.67 | 0.16 | 1.17 | 0.98, 1.41 | 0.09 | 0.51 | 0.37, 0.71 | <0.01 |
| Beta blockers | 1,127 (2.67%) | | 2.15 | 1.65, 2.81 | <0.01 | 1.76 | 1.48, 2.10 | <0.01 | 1.11 | 0.85, 1.46 | 0.43 |
| Statins | 1,299 (3.08%) | | 1.34 | 1.01, 1.77 | 0.04 | 0.93 | 0.78, 1.13 | 0.45 | 1.42 | 1.16, 1.73 | <0.01 |
| Vitamin D | 2,093 (4.96%) | | 1.63 | 1.29, 2.05 | <0.01 | 1.24 | 1.06, 1.46 | 0.01 | 1.52 | 1.27, 1.88 | <0.01 |

Table S4 – Unadjusted HRs for prognostic factors for men with localised disease and unknown location associated with outcomes

HR – Hazard Ratio; BPH – Benign Prostatic Hypertrophy; COPD – Chronic Obstructive Pulmonary Disease; CVA – Cerebrovascular Accident; IHD – Ischaemic Heart Disease; PVD – Peripheral Vascular Disease; T2DM – Type 2 Diabetes Mellitus

For peer review only

| **n = 42, 182** | | | **Prostate cancer mortality** | | | **All-cause mortality** | | | **Systemic therapy** | | |
|---|---|---|---|---|---|---|---|---|---|---|---|
| **Factor** | **Mean (SD)** | **Missing [n (%)]** | **HR per SD[a]** | **95% CI** | **p** | **HR per SD[a]** | **95% CI** | **p** | **HR per SD[a]** | **95% CI** | **p** |
| Age | 76.70 (9.42) | 0 | 1.82 | 1.54, 2.16 | <0.01 | 2.00 | 1.84, 2.19 | <0.01 | 0.96 | 0.90, 1.01 | 0.01 |
| BMI | 27.37 (4.49) | 394 (3.61%) | 1.07 | 0.93, 1.23 | 0.36 | 0.96 | 0.89, 1.03 | 0.28 | 1.04 | 0.99, 1.09 | 0.10 |
| Triglycerides | 1.48 (0.82) | 3,856 (35.37%) | 0.89 | 0.70, 1.12 | 0.31 | 1.02[b] | 0.92, 1.13 | 0.72 | 1.03 | 0.97, 1.09 | 0.38 |
| HDL cholesterol | 1.34 (0.53) | 3,954 (36.27%) | 1.04 | 0.88, 1.23 | 0.67 | 0.97[b] | 0.86, 1.09 | 0.58 | 1.01 | 0.95, 1.07 | 0.79 |
| LDL cholesterol | 2.84 (0.99) | 4,698 (43.10%) | 0.84 | 0.68, 1.03 | 0.09 | 0.90[b] | 0.82, 1.01 | 0.05 | 0.99 | 0.94, 1.05 | 0.81 |
| Hb | 141.81 (16.58) | 2,696 (24.73%) | 0.71 | 0.59, 0.85 | <0.01 | 0.74 | 0.67, 0.80 | <0.01 | 0.90 | 0.85, 0.96 | <0.01 |
| Albumin | 41.41 (4.31) | 2,954 (27.10%) | 0.76 | 0.65, 0.89 | <0.01 | 0.81 | 0.75, 0.88 | <0.01 | 0.92 | 0.87, 0.97 | <0.01 |
| Random glucose | 5.92 (2.11) | 4,525 (41.51%) | 1.28 | 1.08, 1.53 | <0.01 | 1.11 | 0.99, 1.24 | 0.07 | 1.02[b] | 0.95, 1.09 | 0.58 |
| | **Median (IQR)** | **Missing [n (%)]** | | | | | | | | | |
| PSA | 10.4 (6.11, 24.6) | 2,352 (21.58%) | 1.19 | 1.04, 1.35 | 0.01 | 1.14 | 1.02, 1.28 | 0.02 | 1.09 | 0.92, 1.30 | 0.33 |
| CRP | 5 (2, 10) | 8,061 (73.95%) | 1.41 [b] | 1.12, 1.77 | <0.01 | 1.28[b] | 1.11, 1.47 | <0.01 | 1.07 | 0.95, 1.20 | 0.25 |
| Ferritin | 101.5 (45, 197) | 9,495 (87.10%) | 1.80 | 1.08, 3.00 | 0.02 | 1.02[b] | 0.67, 1.56 | 0.92 | 1.06 | 0.85, 1.32 | 0.62 |

Table S5 – Prognostic factors for men with localised disease and unknown location associated with outcomes

[a] Adjusted for age, PSA, Gleason score, TNM stage

[b] Proportional Hazards assumption test not met

HR – Hazard Ratio; SD – Standard Deviation; BMI – Body Mass Index; PSA – Prostate Specific Antigen; HDL – High Density Lipoprotein; LDL – Low Density Lipoprotein; HbA1c – Haemoglobin A1c; CRP – C-Reactive Protein; Hb – Haemoglobin

For peer review only

| n = 42, 182 | | | Prostate cancer mortality | | | All-cause mortality | | | Systemic therapy | | |
|---|---|---|---|---|---|---|---|---|---|---|---|
| **Factor** | **n (%)** | **Missing [n(%)]** | **HR[a]** | **95% CI** | **p** | **HR[a]** | **95% CI** | **p** | **HR[a]** | **95% CI** | **p** |
| Smoker (current/ ex-) | 19,215 (45.56%) | 777 (7.13%) | 1.41 | 1.04, 1.90 | 0.03 | 1.67 | 1.43, 1.96 | <0.01 | 1.22 | 1.10, 1.34 | <0.01 |
| Excess alcohol | 5,926 (14.05%) | 4,370 (40.09%) | 0.65 | 0.40, 1.05 | 0.08 | 0.89[b] | 0.71, 1.13 | 0.35 | 0.99 | 0.86, 1.13 | 0.85 |
| BPH | 4,318 (10.24%) | 3,526 (32.35%) | 0.63 | 0.38, 1.05 | 0.08 | 0.82 | 0.65, 1.03 | 0.08 | 0.73 | 0.62, 0.86 | <0.01 |
| COPD | 3,866 (9.17%) | 3,583 (32.87%) | 0.99 | 0.58, 1.66 | 0.96 | 1.68 | 1.35, 2.09 | <0.01 | 1.18 | 0.99, 1.40 | 0.06 |
| CVA | 2,973 (7.05%) | 3,584 (32.88%) | 0.64 | 0.30, 1.38 | 0.26 | 1.47 | 1.12, 1.93 | <0.01 | 0.90 | 0.71, 1.15 | 0.41 |
| IHD | 7,512 (17.81%) | 3,405 (31.24%) | 1.70 | 1.19, 2.44 | <0.01 | 1.31 | 1.08, 1.58 | <0.01 | 1.01 | 0.87, 1.00 | 0.9 |
| PVD | 1,138 (2.70%) | 3,582 (32.86%) | 2.52 | 1.23, 5.16 | 0.01 | 1.91 | 1.27, 2.85 | <0.01 | 1.09 | 0.75, 1.59 | 0.64 |
| T2DM | 6,233 (14.78%) | 3,448 (31.63%) | 1.06 | 0.72, 1.58 | 0.76 | 0.97 | 0.79, 1.19 | 0.75 | 1.02 | 0.88, 1.17 | 0.83 |
| Aspirin | 2,022 (4.79%) | 16 (0.15%) | 1.55 | 0.79, 3.02 | 0.20 | 1.41 | 0.99, 2.00 | 0.06 | 1.23 | 0.95, 1.60 | 0.12 |
| Metformin | 220 (0.52%) | | | | | 2.76 | 1.03, 7.38 | 0.04 | 1.43 | 0.64, 3.20 | 0.38 |
| Alpha blockers | 1,025 (2.43%) | | 1.28 | 0.59, 2.75 | 0.53 | 1.19 | 0.85, 1.67 | 0.32 | 0.55 | 0.38, 0.79 | <0.01 |
| Beta blockers | 1,127 (2.67%) | | 1.76 | 0.82, 3.75 | 0.15 | 1.82 | 1.27, 2.62 | <0.01 | 1.43 | 1.06, 1.93 | 0.02 |
| Statins | 1,299 (3.08%) | | 1.42 | 0.75, 2.69 | 0.28 | 0.87 | 0.57, 1.32 | 0.51 | 1.09 | 0.86, 1.37 | 0.48 |
| Vitamin D | 2,093 (4.96%) | | 1.13 | 0.55, 2.30 | 0.74 | 1.13 | 0.78, 1.65 | 0.51 | 1.38 | 1.12, 1.72 | <0.01 |

Table S6 – Prognostic factors for men with localised disease and unknown location associated with outcomes

[a] Adjusted for age, PSA, Gleason score, TNM stage

[b] Proportional Hazards assumption test not met

HR – Hazard Ratio; BPH – Benign Prostatic Hypertrophy; COPD – Chronic Obstructive Pulmonary Disease; CVA – Cerebrovascular Accident; IHD – Ischaemic Heart Disease; PVD – Peripheral Vascular Disease; T2DM – Type 2 Diabetes Mellitus

For peer review only

# TRIPOD Checklist: Prediction Model Development and Validation

| Section/Topic | Item | | Checklist Item | Page |
|---|---|---|---|---|
| **Title and abstract** | | | | |
| Title | 1 | D;V | Identify the study as developing and/or validating a multivariable prediction model, the target population, and the outcome to be predicted. | 1 |
| Abstract | 2 | D;V | Provide a summary of objectives, study design, setting, participants, sample size, predictors, outcome, statistical analysis, results, and conclusions. | 3 |
| **Introduction** | | | | |
| Background and objectives | 3a | D;V | Explain the medical context (including whether diagnostic or prognostic) and rationale for developing or validating the multivariable prediction model, including references to existing models. | 4-5 |
| | 3b | D;V | Specify the objectives, including whether the study describes the development or validation of the model or both. | 3, 5 |
| **Methods** | | | | |
| Source of data | 4a | D;V | Describe the study design or source of data (e.g., randomized trial, cohort, or registry data), separately for the development and validation data sets, if applicable. | 6 |
| | 4b | D;V | Specify the key study dates, including start of accrual; end of accrual; and, if applicable, end of follow-up. | 6 |
| Participants | 5a | D;V | Specify key elements of the study setting (e.g., primary care, secondary care, general population) including number and location of centres. | 6 |
| | 5b | D;V | Describe eligibility criteria for participants. | 6 |
| | 5c | D;V | Give details of treatments received, if relevant. | 6 |
| Outcome | 6a | D;V | Clearly define the outcome that is predicted by the prediction model, including how and when assessed. | 6 |
| | 6b | D;V | Report any actions to blind assessment of the outcome to be predicted. | n/a |
| Predictors | 7a | D;V | Clearly define all predictors used in developing the multivariable prediction model, including how and when they were measured. | 6, 8 |
| | 7b | D;V | Report any actions to blind assessment of predictors for the outcome and other predictors. | n/a |
| Sample size | 8 | D;V | Explain how the study size was arrived at. | 7 |
| Missing data | 9 | D;V | Describe how missing data were handled (e.g., complete-case analysis, single imputation, multiple imputation) with details of any imputation method. | 6-7 |
| Statistical analysis methods | 10a | D | Describe how predictors were handled in the analyses. | 6-7 |
| | 10b | D | Specify type of model, all model-building procedures (including any predictor selection), and method for internal validation. | 6-7 |
| | 10c | V | For validation, describe how the predictions were calculated. | n/a |
| | 10d | D;V | Specify all measures used to assess model performance and, if relevant, to compare multiple models. | n/a |
| | 10e | V | Describe any model updating (e.g., recalibration) arising from the validation, if done. | n/a |
| Risk groups | 11 | D;V | Provide details on how risk groups were created, if done. | n/a |
| Development vs. validation | 12 | V | For validation, identify any differences from the development data in setting, eligibility criteria, outcome, and predictors. | n/a |
| **Results** | | | | |
| Participants | 13a | D;V | Describe the flow of participants through the study, including the number of participants with and without the outcome and, if applicable, a summary of the follow-up time. A diagram may be helpful. | 8 |
| | 13b | D;V | Describe the characteristics of the participants (basic demographics, clinical features, available predictors), including the number of participants with missing data for predictors and outcome. | 8, Table 1 |
| | 13c | V | For validation, show a comparison with the development data of the distribution of important variables (demographics, predictors and outcome). | n/a |
| Model development | 14a | D | Specify the number of participants and outcome events in each analysis. | 8 |
| | 14b | D | If done, report the unadjusted association between each candidate predictor and outcome. | Table S1-2 |
| Model specification | 15a | D | Present the full prediction model to allow predictions for individuals (i.e., all regression coefficients, and model intercept or baseline survival at a given time point). | n/a |
| | 15b | D | Explain how to use the prediction model. | n/a |
| Model performance | 16 | D;V | Report performance measures (with CIs) for the prediction model. | n/a |
| Model-updating | 17 | V | If done, report the results from any model updating (i.e., model specification, model performance). | n/a |
| **Discussion** | | | | |
| Limitations | 18 | D;V | Discuss any limitations of the study (such as nonrepresentative sample, few events per predictor, missing data). | 11 |
| Interpretation | 19a | V | For validation, discuss the results with reference to performance in the development data, and any other validation data. | n/a |
| | 19b | D;V | Give an overall interpretation of the results, considering objectives, limitations, results from similar studies, and other relevant evidence. | 9-12 |
| Implications | 20 | D;V | Discuss the potential clinical use of the model and implications for future research. | 12 |
| **Other information** | | | | |
| Supplementary information | 21 | D;V | Provide information about the availability of supplementary resources, such as study protocol, Web calculator, and data sets. | 6 |
| Funding | 22 | D;V | Give the source of funding and the role of the funders for the present study. | 2 |

*Items relevant only to the development of a prediction model are denoted by D, items relating solely to a validation of a prediction model are denoted by V, and items relating to both are denoted D;V. We recommend using the TRIPOD Checklist in conjunction with the TRIPOD Explanation and Elaboration document.

For peer review only - http://bmjopen.bmj.com/site/about/guidelines.xhtml

BMJ Open

# Retrospective cohort study evaluating clinical, biochemical and pharmacological prognostic factors for prostate cancer progression using primary care data

| | |
|---|---|
| Journal: | *BMJ Open* |
| Manuscript ID | bmjopen-2020-044420.R1 |
| Article Type: | Original research |
| Date Submitted by the Author: | 01-Feb-2021 |
| Complete List of Authors: | Merriel, Samuel; University of Exeter, College of Medicine & Health<br>Ingle, Suzanne; University of Bristol School of Social and Community Medicine, Centre for Academic Primary Care<br>May, Margaret; University of Bristol School of Social and Community Medicine,<br>Martin, Richard; University of Bristol, Social Medicine |
| <b>Primary Subject Heading</b>: | Urology |
| Secondary Subject Heading: | Oncology, Epidemiology |
| Keywords: | Prostate disease < UROLOGY, Epidemiology < ONCOLOGY, PRIMARY CARE |
| | |

SCHOLARONE™
Manuscripts

*I, the Submitting Author has the right to grant and does grant on behalf of all authors of the Work (as defined in the below author licence), an exclusive licence and/or a non-exclusive licence for contributions from authors who are: i) UK Crown employees; ii) where BMJ has agreed a CC-BY licence shall apply, and/or iii) in accordance with the terms applicable for US Federal Government officers or employees acting as part of their official duties; on a worldwide, perpetual, irrevocable, royalty-free basis to BMJ Publishing Group Ltd ("BMJ") its licensees and where the relevant Journal is co-owned by BMJ to the co-owners of the Journal, to publish the Work in this journal and any other BMJ products and to exploit all rights, as set out in our licence.*

*The Submitting Author accepts and understands that any supply made under these terms is made by BMJ to the Submitting Author unless you are acting as an employee on behalf of your employer or a postgraduate student of an affiliated institution which is paying any applicable article publishing charge ("APC") for Open Access articles. Where the Submitting Author wishes to make the Work available on an Open Access basis (and intends to pay the relevant APC), the terms of reuse of such Open Access shall be governed by a Creative Commons licence – details of these licences and which Creative Commons licence will apply to this Work are set out in our licence referred to above.*

*Other than as permitted in any relevant BMJ Author's Self Archiving Policies, I confirm this Work has not been accepted for publication elsewhere, is not being considered for publication elsewhere and does not duplicate material already published. I confirm all authors consent to publication of this Work and authorise the granting of this licence.*

For peer review only

# Retrospective cohort study evaluating clinical, biochemical and pharmacological prognostic factors for prostate cancer progression using primary care data

Samuel W D Merriel[1, 2]

Suzanne M Ingle[2]

Margaret T May[2, 3]

Richard M Martin[2, 3]

1 Exeter Medical School, University of Exeter, Exeter, UK

2 Bristol Medical School, Population Health Sciences, University of Bristol, Bristol, UK

3 National Institute for Health Research (NIHR) Bristol Biomedical Research Centre, University Hospitals Bristol NHS Foundation Trust and the University of Bristol, Bristol, UK

**Corresponding author** – Samuel W D Merriel

1.18 College House, Heavitree Road, University of Exeter, Exeter, UK EX1 2LU

+441392 726002

s.w.d.merriel@exeter.ac.uk

For peer review only

# Abstract

**Objectives** – To confirm the association of previously reported prognostic factors with future progression of localised prostate cancer using primary care data and identify new potential prognostic factors for further assessment in prognostic model development and validation.

**Design** – Retrospective cohort study, employing Cox proportional hazards regression controlling for age, PSA, and Gleason score, stratified by diagnostic stage.

**Setting** – Primary care in England

**Participants** – Males with localised prostate cancer diagnosed between 01/01/1987 and 31/12/2016 within the Clinical Practice Research Datalink database, with linked data from the National Cancer Registration and Analysis Service and Office for National Statistics.

**Primary and Secondary outcomes** – Primary outcome measure was prostate cancer mortality. Secondary outcomes measures were all-cause mortality and commencing systematic therapy. Up-staging after diagnosis was not used as a secondary outcome owing to significant missing data.

**Results**

10,901 males (mean age 74.38 +/- 9.03 years) with localised prostate cancer were followed up for a mean of 14.12 (+/- 6.36) years. 2,331 (21.38%) men underwent systemic therapy and 3,250 (31.65%) died, including 1,250 (11.47%) from prostate cancer. Factors associated with an increased risk of prostate cancer mortality included age; high PSA; current or ex-smoker; ischaemic heart disease; high C-Reactive Protein; high ferritin; low haemoglobin; high blood glucose; and low albumin.

**Conclusions**

This study identified several new potential prognostic factors for prostate cancer progression, as well as confirming some known prognostic factors, in an independent primary care data set. Further research is needed to develop and validate a prognostic model for prostate cancer progression.


For peer review only

# Article summary

**Strengths and limitations of this study**

- Large retrospective cohort study of men with localised prostate cancer
- Mean follow-up 14.12 years
- Data available on a wide range of potential prognostic factors for prostate cancer progression
- Missing cancer stage and grade data from NCRAS cancer registry excluded a proportion of the cohort

For peer review only

# Main text

## Introduction

Prostate cancer prognosis and treatment decisions remain a challenging clinical area for clinicians and patients, particularly for men with localised disease at the time of diagnosis. In recent decades, prostate cancer detection rates in many countries have increased markedly, in part as a result of the rising use of asymptomatic prostate specific antigen (PSA) testing[1]. However, more intensive PSA-based detection of prostate cancer has not been convincingly directly correlated with reductions in prostate cancer mortality for all men[2], implying increasing over-detection of clinically insignificant tumours[3]. Treatments for prostate cancer carry a significant risk of morbidity for men[4,5], underlining the importance of being able to identify which men with tumours confined to the prostate at diagnosis are at higher risk of prostate cancer progression and mortality to inform discussions about management options.

Defining and measuring cancer progression with respect to treatment studies is outlined in the Response Evaluation Criteria in Solid Tumours (RECIST) criteria, which was originally published by the World Health Organisation in 2000[6] and most recently updated in 2009[7]. Evidence of tumour shrinkage on imaging and time to development of disease progression are used to measure treatment response. Definitions of cancer progression that are relevant to prognostic studies are less well defined, and numerous clinical, biological and surrogate markers of progression have been proposed in various studies. Prostate cancer mortality appears to be the logical ultimate endpoint of prostate cancer progression, but other measures such as development of metastases[8], biochemical recurrence[9], commencing systemic therapy[10], and protein expression[11] have also been reported.

There are a plethora of prognostic factor studies and prediction tools for prostate cancer risk[12] and prognosis[13] in the published literature. The vast majority are not externally calibrated or validated, and very few are established for use in clinical practice[12]. Initiatives such as the MRC PROGnosis RESearch Strategy Partnership (PROGRESS) partnership highlight the importance of high quality prognostic research to help inform clinical practice[14], and outline methodologically rigorous approaches to achieve this aim[15–17]. Developing clinically useful risk-prediction rules

For peer review only

starts with identifying potentially important prognostic factors which could be incorporated into a prediction model. The aim of the current study is to confirm the association of previously reported prognostic factors with future progression of localised prostate cancer using primary care data and identify new potential prognostic factors for further assessment in prognostic model development and validation.

For peer review only

**Materials and Methods**

The protocol for this study has been published previously in *BMJ Open*[18]. In summary, we undertook a retrospective cohort study using a longitudinal dataset of prospectively collected electronic primary care medical records from General Practices (GPs) in England for the Clinical Practice Research Datalink (CPRD)[19]. This dataset was linked with cancer registry data from the National Cancer Research and Analysis Service (NCRAS)[20] and mortality data from the Office for National Statistics (ONS)[21]. Men were included if they had a diagnosis of prostate cancer entered into their medical record during the 20-year study period (01/01/1987 – 31/12/2016). Localised prostate cancer was defined as T1-2/N0/M0 based on staging data entered into the NCRAS registry, which is determined from a combination of clinical, pathological and radiological data[22].

Potentially relevant clinical, biochemical and pharmacological factors measured in CPRD were identified from a review of the existing published literature (See *BMJ Open* protocol paper[18] for more information about the prognostic factors assessed). The primary outcome of the study was prostate cancer mortality. Secondary outcomes were all-cause mortality and commencing systemic prostate cancer therapy (a measurable proxy for progression and metastatic spread of prostate cancer). Surgery, radiotherapy and brachytherapy were classified as localised therapy, with chemotherapy, hormone treatments (primary or neo adjuvant), and immunotherapy considered systemic therapy. Mortality outcomes were based on primary/immediate cause of death reported in death certification information from the ONS, and therapy outcomes from NCRAS data. In our published protocol[18], up-staging after diagnosis was proposed as a secondary outcome indicating spread of disease; however, this was not used in the final analysis as repeat staging was rarely recorded in the cancer registry.

Descriptive statistics were used to summarise the basic demographic details of the men, and the prevalence of the pre-selected putative prognostic factors. Cox proportional hazards regression was used to estimate crude and mutually adjusted hazard ratios (with 95% confidence intervals) for prostate cancer specific and all-cause mortality according to the prognostic factors, controlling for variables currently used in clinical practice (age, PSA level, Gleason score). Regression analyses

For peer review only - http://bmjopen.bmj.com/site/about/guidelines.xhtml

of continuous prognostic factors were standardised using hazard ratios per change in one standard deviation. A Proportional Hazards test was performed to confirm modelling met regression assumptions. The analysis was also stratified by stage at diagnosis (T1/2N0M0 vs T3+ and/or N1 and/or M1). Sensitivity analysis was performed, assuming all men in the overall sample with unknown tumour location had localised disease. In order to achieve 95% power and detect a difference of 0.1 in prostate cancer mortality for a binary risk factor using an alpha of 0.05, a sample of at least 6,046 men with prostate cancer would be required, assuming that 10% die over a median 10-year follow-up.

This study received ethical approval through the MHRA ISAC process (reference 17_041). The funder had no role in the planning or undertaking of this study, or the preparation of this manuscript.

For peer review only

## Results

54,500 men within CPRD had a diagnosis of prostate cancer entered into their primary care medical record during the study period. Baseline participant data are shown in Table 1. Tumour-Node-Metastases (TNM) staging data from the linked cancer registry were available for 7,646 (14.03%) of the sample population and treatment data were available for 22,766 (41.77%) men. Missing TNM staging data from the cancer registry was lower for men diagnosed in more recent years: there were no TNM stage data for men diagnosed before 1993, rising to 37.2% with TNM stage data (1,064/2,836) in 2015. This is consistent with a recent validation study of the NCRAS prostate cancer registry that showed low levels of completeness of TNM stage and Gleason score data prior to 2010[23]. Using the available staging and treatment data, 10,901 (20%) men were identified as having localised prostate cancer at the time of diagnosis and were included in the final cohort for analysis, with a mean follow-up of 14.12 (+/-6.36) years. Levels of missing data for selected prognostic factors within CPRD varied.

1,250 men with localised disease died of prostate cancer over the course of follow-up, giving a prostate cancer mortality rate of 8.1 per 1,000 person-years. The total number of deaths for included men was 3,250 (21.11 deaths per 1,000 person-years). 2,331 (21.38%) men with localised disease received systemic therapy in the follow-up period after diagnosis. For over 90% of the men it was unknown whether they were re-investigated for cancer staging after diagnosis or not (see Table 2).

Raised acute phase reactants (C-Reactive Protein [CRP] [adjusted HR per SD 1.35 95% CI 1.02, 1.77]), ferritin (adjusted HR per SD 2.03 95% CI 1.21, 3.39) and random glucose (adjusted HR per SD 1.27 95% CI 1.06, 1.54) were associated with prostate cancer mortality. Anaemia (adjusted HR per SD 0.72 95% CI 0.59, 0.88) and low albumin (adjusted HR per SD 0.81 95% CI 0.67, 0.97) were also associated with this outcome. No medications assessed were associated with prostate cancer mortality. Current and ex-smokers (adjusted HR 1.47 95% CI 1.05, 2.05), and patients with a history of ischaemic heart disease (adjusted HR 1.79 95% CI 1.20, 2.66) had a higher risk of prostate cancer mortality over the study period.

For peer review only

Raised CRP, anaemia, and low albumin were biochemical factors associated with all-cause mortality; with anaemia and low albumin also being associated with commencing systemic therapy. A number of other factors were also associated with all-cause mortality, including age, raised PSA, smoking and smoking-related disease, cardiovascular diseases, as well as current use of aspirin or beta blockers. Smoking and beta blockers were also associated with increased risk of systemic therapy, as were vitamin D supplements. Benign prostatic hyperplasia and alpha blocker prescription were associated with a reduced risk of commencing systemic therapy (See Tables 3 & 4 for adjusted analysis results, and Supplementary Tables S1 & S2 for unadjusted results).

Sensitivity analysis including all participants with unknown tumour location showed a relationship between a history of stroke and all-cause mortality (adjusted HR 1.47 95% CI 1.12, 1.93 p = 0.006). The relationship between aspirin and prostate cancer mortality altered to very weak evidence for association (adjusted HR 1.55 95% CI 0.79, 3.02 p = 0.2). For all other factors measured and for all three outcomes in the analysis, the direction of relationship did not change and the magnitude of relationship stayed relatively stable (see Supplementary Tables S3-6).

For peer review only

## Discussion

This retrospective cohort study utilised primary care medical records data for men with localised prostate cancer from CPRD to confirm prognostic factors associated with prostate cancer progression. Well-known factors already incorporated into clinical guidelines, such as age and PSA, were confirmed as being individual prognostic factors. In addition, further clinical (history of smoking or ischaemic heart disease) and biochemical (anaemia or high ferritin) factors were found to be strongly associated with prostate cancer mortality. Anaemia, low albumin, raised PSA, history of ischaemic heart disease, and smoking were also strongly associated with all-cause mortality, as were peripheral vascular disease, COPD, and beta blocker use. Smoking history was strongly associated with future systemic therapy, as were recent prescriptions of alpha blockers, or vitamin D supplements.

This analysis confirms the prognostic associations of some factors in prostate cancer progression. Smoking has also been found to be a risk factor for prostate cancer progression and mortality in cohort studies[24] and systematic reviews[8]. Low albumin was associated with prostate cancer mortality in the AMORIS cohort[25] and, along with anaemia[26], is a more widely accepted predictor of poor cancer outcomes[27]. The published literature around the prognostic effect of beta blockers for prostate cancer patients has been more mixed[28], with this study lending weight to the evidence of increased mortality in cancer patients. BMI was not shown to be associated with prostate cancer and overall mortality in this study. Whilst some observational studies of prostate cancer have suggested an association may exist[8,29,30], reviews of trial data have demonstrated higher BMI may actually improve the prognosis for men with cancer[31].

This study attempted to confirm prognostic factors in a primary care dataset that could be used in a model to predict prostate cancer progression at the time of diagnosis, prior to any treatment being initiated. This approach could allow the identified prognostic factors to be used to develop a new prognostic tool to inform treatment decisions between a patient and their treating team. There are already examples of similar prognostic tools available for use, including Predict Prostate (https://prostate.predict.nhs.uk/). However, these tools have only been developed using secondary care data[32], which may not capture all important prognostic factors or have

For peer review only - http://bmjopen.bmj.com/site/about/guidelines.xhtml

equivalent length of follow-up of patients in their development or calibration cohorts. In the context of on-going challenges with prognostication for men with localised prostate cancer, and the increasing numbers of men being diagnosed every year, getting the most accurate information to inform treatment discussions between patients and their treating physicians is vital.

*Strengths and limitations*

This study has a number of unique features. This is the first study that the authors are aware of to utilise a primary care dataset to identify and confirm prognostic factors associated with prostate cancer progression. CPRD contains all data held in the primary care records of millions of UK patients, allowing the inclusion of a range of potentially important prognostic factors. Using a primary care dataset from the NHS also provided long-term data for included patients, with a mean follow-up of over 14 years. Prolonged follow-up for men with prostate cancer is important as many patients can live for years before their cancer progresses. The lack of high quality prognostic research discussed in the introduction is not limited to prostate cancer, with many other prognostic factor studies being conducted in similarly flawed ways[33–35]. This study sought to take a confirmatory approach to postulated prognostic factors in prostate cancer in a rigorous manner, following the methodological recommendations of the REMARK guidelines[36] and the PROGRESS partnership[14–17].

There are some limitations of this study that need to be considered. Previous research has shown that the prostate cancer registry in England has strong case completeness, but significant missing TNM stage and Gleason score data up until recent years[23]. Data completeness and quality within NCRAS continues to improve, and there is no equivalent UK cancer registry dataset with more complete data available at this present time[22]. This level of missing data meant it was unknown whether the majority of potentially included men had localised disease or not. Even so, the study was still powered to answer the research question, and sensitivity analyses showed minimal changes to almost all relationships between the prognostic factors of interest and the study outcomes. Misattribution of prostate cancer as the primary cause of death may occur in some frail, elderly patients or patients with multimorbidity, affecting the primary outcome of this study. There is evidence of misattribution of prostate cancer as a cause of death in other high-

For peer review only - http://bmjopen.bmj.com/site/about/guidelines.xhtml

income countries[37,38], however an English study comparing death certification to a blinded, independent panel showed that ONS data on prostate cancer mortality classification is highly accurate[39]. This study uses a retrospective design interrogating electronic primary care records. It relies on accurate coding from GPs[40], and there was significant missing data for some prognostic factors.

This study took a confirmatory approach to identify which prognostic factors for prostate cancer progression may be relevant, and some new prognostic factors not currently recommended for use in clinical practice were identified. These prognostic factors could be used to generate a more robust clinical risk prediction tool to guide treatment decision-making. Developing an accurate prediction tool for prostate cancer progression, not just mortality, could be more useful for informing management discussions between patients and clinicians.

For peer review only

**Ethical approval**

This study received ethical approval from the Independent Scientific Advisory Committee (ISAC) of the Medicines and Healthcare products Regulatory Authority (MHRA) – Protocol reference 17_041. It was conducted in accordance with the Declaration of Helsinki.

**Funding statement**

SWDM is supported by the Can Test Collaborative, which is funded by CRUK (C8640/A23385). This work was supported from an Academic Clinical Fellowship in Primary Care for SWDM, funded by the National Institute for Health Research and Health Education England. The views expressed in this publication are those of the author(s) and not necessarily those of the NHS, the National Institute for Health Research, Health Education England or the Department of Health. RMM was supported by a CRUK programme grant, the Integrative Cancer Epidemiology Programme (C18281/A19169). MTM was supported by the NIHR Biomedical Research Centre at University Hospitals Bristol NHS Foundation Trust and the University of Bristol.

**Author statement**

SWDM conceived and designed the work that has led to this submission. He acquired the data and performed the analysis. He drafted the manuscript and approves the final version. He agrees to be accountable for all aspects of the work. As corresponding author, he also confirms he has full access to the data in the study and has taken final responsibility for the decision to submit for publication.

SMI played an important role in the data analysis and interpretation of the results. She revised the manuscript and approved the final version. She agrees to be accountable for all aspects of the work.

MTM helped design the work that has led to this submission, and supported interpretation of the results. She also provided study supervision to SWDM. She has revised the manuscript and approved the final version. She agrees to be accountable for all aspects of the work.

RMM helped to conceive and design the work that has led to this submission. He also provided study supervision to SWDM He has revised the manuscript and approved the final version. He agrees to be accountable for all aspects of the work.

For peer review only

**Conflict of interest disclosure statement** – The authors declare no potential conflicts of interest

**Data statement**

This study analysed a CPRD dataset, with linked NCRAS and ONS data. Permission was not sought to share the dataset publicly.

**Patient and Public Involvement**

Patients and the public were not involved in the design or conduct of this research.

## References


1 Collin SM, Martin RM, Metcalfe C, *et al.* Prostate-cancer mortality in the USA and UK in 1975-2004: an ecological study. *Lancet Oncol* 2008;**9**:445–52. doi:10.1016/S1470-2045(08)70104-9

2 Ferlay J, Soerjomataram I, Ervik M, *et al.* GLOBOCAN 2012 v1.0, Cancer Incidence and Mortality Worldwide: IARC CancerBase No. 11 [Internet]. Int. Agency Res. Cancer. 2013.http://globocan.iarc.fr (accessed 24 May 2018).

3 Sandhu GS, Andriole GL. Overdiagnosis of prostate cancer. *J Natl Cancer Inst - Monogr* 2012;**2012**:146–51. doi:10.1093/jncimonographs/lgs031

4 Donovan JL, Hamdy FC, Lane JA, *et al.* Patient-Reported Outcomes after Monitoring, Surgery, or Radiotherapy for Prostate Cancer. *N Engl J Med* 2016;**375**:1425–37. doi:10.1056/NEJMoa1606221

5 Hamdy FC, Donovan JL, Lane JA, *et al.* 10-Year Outcomes after Monitoring, Surgery, or Radiotherapy for Localized Prostate Cancer. *N Engl J Med* 2016;:NEJMoa1606220-10.http://www.nejm.org/doi/10.1056/NEJMoa1606220

6 Therasse P, Arbuck SG, Eisenhauer EA, *et al.* New guidelines to evaluate the response to treatment in solid tumors. European Organization for Research and Treatment of Cancer, National Cancer Institute of the United States, National Cancer Institute of Canada. JNCI J. Natl. Cancer Inst. 2000;**92**:205–16.http://jnci.oxfordjournals.org/cgi/doi/10.1093/jnci/92.3.205


For peer review only

7 Eisenhauer EA, Therasse P, Bogaerts J, *et al.* New response evaluation criteria in solid tumours: Revised RECIST guideline (version 1.1). 2009;**45**:228–47.http://dx.doi.org/10.1016/j.ejca.2008.10.026

8 Peisch SF, Blarigan EL Van, Chan JM, *et al.* Prostate cancer progression and mortality: a review of diet and lifestyle factors. *World J Urol* 2017;**35**:867–74. doi:10.1007/s00345-016-1914-3.Prostate

9 Bianco-Miotto T, Chiam K, Buchanan G, *et al.* Global levels of specific histone modifications and an epigenetic gene signature predict prostate cancer progression and development. *Cancer Epidemiol Biomarkers Prev* 2010;**19**:2611–22. doi:10.1158/1055-9965.EPI-10-0555

10 Fleshner N, Lucia MS, Melich K, *et al.* Effect of dutasteride on prostate cancer progression and cancer diagnosis on rebiopsy in the REDEEM active surveillance study. *J Clin Oncol* 2011;**29**:no pagination. doi:10.1200/jco.2011.29.7

11 Cullen J, Young D, Chen Y, *et al.* Predicting Prostate Cancer Progression as a Function of ETS-related Gene Status, Race, and Obesity in a Longitudinal Patient Cohort. *Eur Urol Focus* 2017;**1**:1–7. doi:10.1016/j.euf.2017.02.016

12 Louie KS, Seigneurin A, Cathcart P, *et al.* Do prostate cancer risk models improve the predictive accuracy of PSA screening? A meta-analysis. *Ann Oncol* 2015;**26**:848–64. doi:10.1093/annonc/mdu525

13 Shariat SF, Kattan MW, Vickers AJ, *et al.* Critical review of prostate cancer predictive tools. *Futur Oncol* 2009;**5**:1555–84.http://www.futuremedicine.com/doi/10.2217/fon.09.121

14 Hemingway H, Croft P, Perel P, *et al.* Prognosis research strategy (PROGRESS) 1: A framework for researching clinical outcomes. *BMJ* 2013;**346**:e5595–e5595.http://www.bmj.com/cgi/doi/10.1136/bmj.e5595

15 Riley RD, Hayden JA, Steyerberg EW, *et al.* Prognosis Research Strategy (PROGRESS) 2: Prognostic Factor Research. *PLoS Med* 2013;**10**:e1001380-9.http://dx.plos.org/10.1371/journal.pmed.1001380

16 Steyerberg EW, Moons KGM, van der Windt DA, *et al.* Prognosis Research Strategy (PROGRESS) 3: Prognostic Model Research. *PLoS Med* 2013;**10**:e1001381-9.http://dx.plos.org/10.1371/journal.pmed.1001381

17 Hingorani AD, Windt DA v d, Riley RD, *et al.* Prognosis research strategy (PROGRESS) 4:

For peer review only

Stratified medicine research. *BMJ* 2013;**346**:e5793–e5793.http://www.bmj.com/cgi/doi/10.1136/bmj.e5793

18 Merriel SWD, May MT, Martin RM. Predicting prostate cancer progression: protocol for a retrospective cohort study to identify prognostic factors for prostate cancer outcomes using routine primary care data. *BMJ Open* 2018;**8**:e019409. doi:10.1136/ bmjopen-2017-019409

19 CPRD. Welcome to the Clinical Practice Research Datalink. https://www.cprd.com/home/

20 NCRAS. National Cancer Research and Analysis Service. papers3://publication/uuid/CBEF1C52-AEBE-4B92-A099-2BC799944207

21 ONS. Deaths Registered in England and Wales (Series DR), 2016. London: 2017. http://www.ons.gov.uk/ons/rel/vsob1/mortality-statistics--deaths-registered-in-england-and-wales--series-dr-/2014/stb-mortality-stats-2014.html

22 Henson KE, Elliss-Brookes L, Coupland VH, *et al.* Data Resource Profile: National Cancer Registration Dataset in England. *Int J Epidemiol* 2020;**49**:16-16h. doi:10.1093/ije/dyz076

23 Merriel SWD, Turner EL, Walsh E, *et al.* Cross-sectional study evaluating data quality of the National Cancer Registration and Analysis Service (NCRAS) prostate cancer registry data using the Cluster randomised trial of PSA testing for Prostate cancer (CAP). *BMJ Open* 2017;**7**:e015994. doi:10.1136/bmjopen-2017-015994

24 Rohrmann S, Genkinger JM, Burke A, *et al.* Smoking and Risk of Fatal Prostate Cancer in a Prospective U.S. Study. *Urology* 2007;**69**:721–5.http://linkinghub.elsevier.com/retrieve/pii/S0090429506026446

25 Arthur R, Williams R, Garmo H, *et al.* Serum inflammatory markers in relation to prostate cancer severity and death in the Swedish AMORIS study. *Int J Cancer* 2018;**142**:2254–62. doi:10.1002/ijc.31256

26 Van Belle SJP. What is the value of hemoglobin as a prognostic and predictive factor in cancer? *Eur J Cancer, Suppl* 2004;**2**:11–9. doi:10.1016/S1359-6349(03)00103-4

27 Gupta D, Lis CG. Pretreatment serum albumin as a predictor of cancer survival: A systematic review of the epidemiological literature. *Nutr J* 2010;**9**:1–16. doi:10.1186/1475-2891-9-69

28 Assayag J, Pollak MN, Azoulay L. Post-diagnostic use of beta-blockers and the risk of death in patients with prostate cancer. *Eur J Cancer* 2014;**50**:2838–45.http://dx.doi.org/10.1016/j.ejca.2014.08.006

For peer review only

29 Cantarutti A, Bonn SE, Adami H-O, *et al.* Body mass index and mortality in men with prostate cancer. *Prostate* 2015;**75**:1129–36. doi:10.1002/pros.23001

30 Haque R, Van Den Eeden SK, Wallner LP, *et al.* Association of body mass index and prostate cancer mortality. *Obes Res Clin Pract* 2014;**8**:e374-81. doi:10.1016/j.orcp.2013.06.002

31 Greenlee H, Unger JM, LeBlanc M, *et al.* Association between body mass index and cancer survival in a pooled analysis of 22 clinical trials. *Cancer Epidemiol Biomarkers Prev* 2017;**26**:21–9. doi:10.1158/1055-9965.EPI-15-1336

32 Gnanapragasam VJ, Lophatananon A, Wright KA, *et al.* Improving Clinical Risk Stratification at Diagnosis in Primary Prostate Cancer: A Prognostic Modelling Study. *PLoS Med* 2016;**13**:1–18. doi:10.1371/journal.pmed.1002063

33 Kyzas PA, Denaxa-Kyza D, Ioannidis JPA. Almost all articles on cancer prognostic markers report statistically significant results. *Eur J Cancer* 2007;**43**:2559–79. doi:10.1016/j.ejca.2007.08.030

34 Kyzas PA, Loizou KT, Ioannidis JPA. Selective reporting biases in cancer prognostic factor studies. *J Natl Cancer Inst* 2005;**97**:1043–55. doi:10.1093/jnci/dji184

35 Hemingway H, Riley RD, Altman DG. Ten steps towards improving prognosis research. *BMJ* 2010;**340**:410–4. doi:10.1136/bmj.b4184

36 McShane LM, Altman DG, Sauerbrei W, *et al.* Reporting Recommendations for Tumor Marker Prognostic Studies (REMARK). *JNCI J Natl Cancer Inst* 2005;**97**:1180–4.https://academic.oup.com/jnci/article-lookup/doi/10.1093/jnci/dji237

37 Moghanaki D, Howard LE, De Hoedt A, *et al.* Validity of the National Death Index to ascertain the date and cause of death in men having undergone prostatectomy for prostate cancer. *Prostate Cancer Prostatic Dis* 2019;**22**:633–5. doi:10.1038/s41391-019-0146-1

38 Löffeler S, Halland A, Weedon-Fekjær H, *et al.* High Norwegian prostate cancer mortality: evidence of over-reporting. *Scand J Urol* 2018;**52**:122–8. doi:10.1080/21681805.2017.1421260

39 Turner EL, Metcalfe C, Donovan JL, *et al.* Contemporary accuracy of death certificates for coding prostate cancer as a cause of death: Is reliance on death certification good enough? A comparison with blinded review by an independent cause of death evaluation committee. *Br J Cancer* 2016;**115**:90–4.http://dx.doi.org/10.1038/bjc.2016.162

For peer review only

40 Reeves D, Springate DA, Ashcroft DM, *et al.* Can analyses of electronic patient records be independently and externally validated? The effect of statins on the mortality of patients with ischaemic heart disease: a cohort study with nest case-control analysis. *BMJ Open* 2014;**4**:1–11.http://dx.doi.org/10.1136/bmjopen-2014-004952

For peer review only

| | Localised n = 10,901 | Missing data |
|---|---|---|
| Mean (SD) | | |
| Age (yrs) | 74.38 (+/- 9.03) | 0% |
| BMI (kg/m$^2$) | 27.43 (+/- 4.48) | 5.64% |
| Follow-up (yrs) | 14.12 (+/- 6.36) | 0% |
| Median (IQR) | | |
| PSA (ng/mL) | 8.4 (5.55, 14.6) | 30.66% |
| n (%) | | |
| Gleason score | | |
| 6 | 3,655 (33.53%) | 33.23% |
| 7+ | 4,420 (40.55%) | |
| Family history of prostate cancer | 70 (0.64%) | 55.11% |
| Ethnicity | | |
| White | 7,361 (67.53%) | 29.79% |
| Mixed | 21 (0.19%) | |
| Asian | 75 (0.69%) | |
| Black | 156 (1.43%) | |
| Other | 41 (0.38%) | |

Table 1 – Baseline participant data

For peer review only

| | | Prostate cancer mortality | All-cause mortality | Systemic therapy | Upstaging[a] |
|---|---|---|---|---|---|
| Included | Localised (T1/2 N0 M0) n = 10,901 | 1,250 (11.47%) | 3,450 (31.65%) | 2,331 (21.38%) | 45 (0.41%) |
| Excluded | Invasive (T3+ / N1 / M1) n = 12,318 | 3,894 (31.61%) | 6,916 (56.15%) | 10,881 (88.33%) | 28 (0.23%) |
| Excluded | Unknown n = 31,281 | 1,540 (4.92%) | 5,420 (17.33%) | 31,954 (58.63%) | 19 (0.06%) |

Table 2 – Primary and Secondary outcomes for included and excluded participants

[a] Repeat staging data missing for 50,119 (91.96%) of sample

For peer review only

| n = 10,901 | | | Prostate cancer mortality | | | All-cause mortality | | | Systemic therapy | | |
|---|---|---|---|---|---|---|---|---|---|---|---|
| **Factor** | **Mean (SD)** | **Missing [n (%)]** | **HR per SD[a]** | **95% CI** | **p** | **HR per SD[a]** | **95% CI** | **p** | **HR per SD[a]** | **95% CI** | **p** |
| Age | 74.39 (9.03) | 0 | 1.70 | 1.40, 2.06 | <0.01 | 1.92 | 1.74, 2.12 | <0.01 | 1 | 0.95, 1.06 | 0.98 |
| BMI | 27.43 (4.48) | 394 (3.61%) | 1.05 | 0.90, 1.08 | 0.52 | 0.97 | 0.90, 1.05 | 0.51 | 1.04 | 0.99, 1.09 | 0.10 |
| Triglycerides | 1.45 (0.80) | 3,856 (35.37%) | 0.83 | 0.64, 1.08 | 0.16 | 1.00[b] | 0.90, 1.13 | 0.93 | 1.03 | 0.97, 1.09 | 0.37 |
| HDL cholesterol | 1.35 (0.43) | 3,954 (36.27%) | 1.05 | 0.89, 1.23 | 0.56 | 1.01 [b] | 0.91, 1.12 | 0.86 | 1.01 | 0.95, 1.07 | 0.75 |
| LDL cholesterol | 2.95 (0.99) | 4,698 (43.10%) | 0.86 | 0.69, 1.07 | 0.18 | 0.92 [b] | 0.82, 1.02 | 0.12 | 0.99 | 0.94, 1.05 | 0.86 |
| Hb | 144.28 (14.35) | 2,696 (24.73%) | 0.72 | 0.59, 0.88 | <0.01 | 0.74 | 0.67, 0.82 | <0.01 | 0.92 | 0.86, 0.98 | 0.01 |
| Albumin | 41.83 (3.94) | 2,954 (27.10%) | 0.81 | 0.67, 0.97 | 0.02 | 0.83 | 0.76, 0.91 | <0.01 | 0.94 | 0.89, 0.99 | 0.04 |
| Random glucose | 5.70 (2.11) | 4,525 (41.51%) | 1.27 | 1.06, 1.54 | 0.01 | 1.12 | 0.99, 1.25 | 0.06 | 1.02[b] | 0.95, 1.09 | 0.66 |
| | **Median (IQR)** | **Missing [n (%)]** | | | | | | | | | |
| PSA | 8.4 (5.55, 14.60) | 2,352 (21.58%) | 1.71 | 1.32, 2.23 | <0.01 | 1.46 | 1.19, 1.78 | <0.01 | 1.34 | 1.06, 1.68 | 0.01 |
| CRP | 3.9 (2, 8) | 8,061 (73.95%) | 1.35 [b] | 1.02, 1.77 | 0.03 | 1.23 [b] | 1.05, 1.45 | 0.01 | 1.07 | 0.95, 1.20 | 0.24 |
| Ferritin | 108.6 (47, 196) | 9,495 (87.10%) | 2.03 | 1.21, 3.39 | <0.01 | 0.98 [b] | 0.60, 1.59 | 0.93 | 1.05 | 0.85, 1.31 | 0.64 |

1 Table 3 – Prognostic factors for men with localised disease associated with outcomes

2 [a] Adjusted for age, PSA, Gleason score, TNM stage

3 [b] Proportional Hazards assumption test not met

4 HR – Hazard Ratio; SD – Standard Deviation; BMI – Body Mass Index; PSA – Prostate Specific Antigen; HDL – High Density Lipoprotein; LDL – Low Density

5 Lipoprotein; HbA1c – Haemoglobin A1c; CRP – C-Reactive Protein; Hb – Haemoglobin

6

For peer review only

| n = 10,901 | | | Prostate cancer mortality | | | All-cause mortality | | | Systemic therapy | | |
|---|---|---|---|---|---|---|---|---|---|---|---|
| **Factor** | **n (%)** | **Missing [n(%)]** | **HR[a]** | **95% CI** | **p** | **HR[a]** | **95% CI** | **p** | **HR[a]** | **95% CI** | **p** |
| Smoker (current/ ex-) | 5,112 (46.89%) | 777 (7.13%) | 1.47 | 1.05, 2.05 | 0.02 | 1.66 | 1.39, 1.98 | <0.01 | 1.21 | 1.09, 1.33 | <0.01 |
| Excess alcohol | 1,829 (16.78%) | 4,370 (40.09%) | 0.61 | 0.36, 1.04 | 0.07 | 0.91[b] | 0.71, 1.18 | 0.47 | 0.99 | 0.87, 1.13 | 0.88 |
| BPH | 1,169 (10.72%) | 3,526 (32.35%) | 0.64 | 0.36, 1.11 | 0.11 | 0.81 | 0.62, 1.05 | 0.11 | 0.76 | 0.65, 0.90 | <0.01 |
| COPD | 862 (7.91%) | 3,583 (32.87%) | 0.86 | 0.47, 1.57 | 0.63 | 1.64 | 1.29, 2.09 | <0.01 | 1.18 | 0.99, 1.41 | 0.06 |
| CVA | 553 (5.07%) | 3,584 (32.88%) | 0.90 | 0.42, 1,94 | 0.79 | 1.19 | 0.85, 1.68 | 0.30 | 0.92 | 0.72, 1.17 | 0.49 |
| IHD | 1,548 (14.20%) | 3,405 (31.24%) | 1.79 | 1.20, 2.66 | <0.01 | 1.25 | 1.02, 1.55 | 0.04 | 1.01 | 0.87, 1.18 | 0.86 |
| PVD | 202 (1.85%) | 3,582 (32.86%) | 2.24 | 0.98, 5.12 | 0.06 | 1.91 | 1.24, 2.95 | <0.01 | 1.04 | 0.71, 1.51 | 0.85 |
| T2DM | 1,508 (13.83%) | 3,448 (31.63%) | 0.97 | 0.62, 1.51 | 0.89 | 0.95 | 0.76, 1.19 | 0.68 | 0.99 | 0.86, 1.14 | 0.91 |
| Aspirin | 426 (3.91%) | 16 (0.15%) | 1.88 | 0.96, 3.70 | 0.06 | 1.58 | 1.09, 2.29 | 0.02 | 1.24 | 0.95, 1.60 | 0.11 |
| Metformin | 33 (0.30%) | | | | | 2.74 | 0.88, 8.49 | 0.08 | 1.73 | 0.77, 3.85 | 0.18 |
| Alpha blockers | 305 (2.80%) | | 1.55 | 0.72, 3.35 | 0.26 | 1.15 | 0.76, 1.73 | 0.52 | 0.57 | 0.39, 0.82 | <0.01 |
| Beta blockers | 265 (2.43%) | | 2.03 | 0.89, 4.60 | 0.09 | 1.79 | 1.18, 2.72 | <0.01 | 1.48 | 1.09, 1.99 | 0.01 |
| Statins | 339 (3.11%) | | 1.65 | 0.87, 3.15 | 0.13 | 1.01 | 0.66, 1.53 | 0.97 | 1.06 | 0.84, 1.34 | 0.61 |
| Vitamin D | 465 (4.27%) | | 1.33 | 0.65, 2.71 | 0.44 | 1.13 | 0.78, 1.65 | 0.51 | 1.35 | 1.09, 1.68 | <0.01 |

Table 4 – Prognostic factors for men with localised disease associated with outcomes

[a] Adjusted for age, PSA, Gleason score, TNM stage

[b] Proportional Hazards assumption test not met

HR – Hazard Ratio; BPH – Benign Prostatic Hypertrophy; COPD – Chronic Obstructive Pulmonary Disease; CVA – Cerebrovascular Accident; IHD – Ischaemic Heart Disease; PVD – Peripheral Vascular Disease; T2DM – Type 2 Diabetes Mellitus

For peer review only

| n = 10,901 | | | Prostate cancer mortality | | | All-cause mortality | | | Systemic therapy | | |
|---|---|---|---|---|---|---|---|---|---|---|---|
| **Factor** | **Mean (SD)** | **Missing [n (%)]** | **HR per SD** | **95% CI** | **p** | **HR per SD** | **95% CI** | **p** | **HR per SD** | **95% CI** | **p** |
| Age | 74.39 (9.03) | 0 | 1.66 | 1.56, 1.76 | <0.01 | 1.82 | 1.76, 1.89 | <0.01 | 0.81 | 0.77, 0.84 | <0.01 |
| BMI | 27.43 (4.48) | 394 (3.61%) | 0.96 | 0.91, 1.02 | 0.19 | 0.97 | 0.94, 1.01 | 0.15 | 1.07 | 1.03, 1.12 | <0.01 |
| Triglycerides | 1.45 (0.80) | 3,856 (35.37%) | 0.93 | 0.84, 1.02 | 0.12 | 0.97 | 0.92, 1.02 | 0.28 | 0.99 | 0.95, 1.04 | 0.82 |
| HDL cholesterol | 1.35 (0.43) | 3,954 (36.27%) | 0.98 | 0.88, 1.09 | 0.66 | 0.98 | 0.92, 1.04 | 0.49 | 1.03 | 0.98, 1.08 | 0.23 |
| LDL cholesterol | 2.95 (0.99) | 4,698 (43.10%) | 0.90 | 0.81, 0.99 | 0.03 | 0.87 | 0.82, 0.92 | <0.01 | 0.95 | 0.91, 0.99 | 0.03 |
| Hb | 144.28 (14.35) | 2,696 (24.73%) | 0.55 | 0.51, 0.58 | <0.01 | 0.62 | 0.59, 0.64 | <0.01 | 0.95 | 0.89, 0.99 | 0.03 |
| Albumin | 41.83 (3.94) | 2,954 (27.10%) | 0.71 | 0.67, 0.74 | <0.01 | 0.74 | 0.71, 0.77 | <0.01 | 0.96 | 0.91, 1.00 | 0.06 |
| Random glucose | 5.70 (2.11) | 4,525 (41.51%) | 1.18 | 1.10, 1.27 | <0.01 | 1.14 | 1.09, 1.20 | <0.01 | 1.02 | 0.97, 1.07 | 0.48 |
| | **Median (IQR)** | **Missing [n (%)]** | | | | | | | | | |
| PSA | 8.4 (5.55, 14.60) | 2,352 (21.58%) | 1.45 | 1.39, 1.51 | <0.01 | 1.36 | 1.31, 1.41 | <0.01 | 1.01 | 0.86, 1.18 | 0.90 |
| CRP | 3.9 (2, 8) | 8,061 (73.95%) | 1.27 | 1.19, 1.36 | <0.01 | 1.22 | 1.16, 1.28 | <0.01 | 1.01 | 0.92, 1.10 | 0.90 |
| Ferritin | 108.6 (47, 196) | 9,495 (87.10%) | 1.48 | 1.25, 1.75 | <0.01 | 1.13 | 0.97, 1.31 | 0.11 | 1.02 | 0.89, 1.16 | 0.79 |

Table S1 – Unadjusted HRs for prognostic factors for men with localised disease associated with outcomes

HR – Hazard Ratio; SD – Standard Deviation; BMI – Body Mass Index; PSA – Prostate Specific Antigen; HDL – High Density Lipoprotein; LDL – Low Density Lipoprotein; HbA1c – Haemoglobin A1c; CRP – C-Reactive Protein; Hb – Haemoglobin

For peer review only

| n = 10,901 | | | Prostate cancer mortality | | | All-cause mortality | | | Systemic therapy | | |
|---|---|---|---|---|---|---|---|---|---|---|---|
| **Factor** | **n (%)** | **Missing [n(%)]** | **HR** | **95% CI** | **p** | **HR** | **95% CI** | **p** | **HR** | **95% CI** | **p** |
| Smoker (current/ ex-) | 5,112 (46.89%) | 777 (7.13%) | 1.24 | 1.09, 1.40 | <0.01 | 1.36 | 1.26, 1.46 | <0.01 | 1.20 | 1.10, 1.30 | <0.01 |
| Excess alcohol | 1,829 (16.78%) | 4,370 (40.09%) | 0.75 | 0.62, 0.91 | <0.01 | 0.67 | 0.60, 0.74 | <0.01 | 0.92 | 0.80, 1.06 | 0.26 |
| BPH | 1,169 (10.72%) | 3,526 (32.35%) | 0.61 | 0.49, 0.76 | <0.01 | 0.79 | 0.70, 0.88 | <0.01 | 0.70 | 0.61, 0.80 | <0.01 |
| COPD | 862 (7.91%) | 3,583 (32.87%) | 0.95 | 0.77, 1.18 | 0.67 | 1.41 | 1.26, 1.58 | <0.01 | 1.08 | 0.94, 1.25 | 0.26 |
| CVA | 553 (5.07%) | 3,584 (32.88%) | 1.48 | 1.18, 1.85 | <0.01 | 1.89 | 1.67, 2.13 | <0.01 | 0.78 | 0.64, 0.96 | 0.02 |
| IHD | 1,548 (14.20%) | 3,405 (31.24%) | 1.62 | 1.39, 1.88 | <0.01 | 1.79 | 1.64, 1.95 | <0.01 | 0.89 | 0.79, 1.00 | 0.05 |
| PVD | 202 (1.85%) | 3,582 (32.86%) | 1.80 | 1.31, 2.49 | <0.01 | 1.87 | 1.55, 2.25 | <0.01 | 0.84 | 0.62, 1.14 | 0.26 |
| T2DM | 1,508 (13.83%) | 3,448 (31.63%) | 1.13 | 0.96, 1.33 | 0.16 | 1.03 | 0.94, 1.14 | 0.49 | 0.93 | 0.83, 1.04 | 0.20 |
| Aspirin | 426 (3.91%) | 16 (0.15%) | 2.33 | 1.88, 2.90 | <0.01 | 2.18 | 1.90, 2.49 | <0.01 | 1.24 | 0.99, 1.54 | 0.06 |
| Metformin | 33 (0.30%) | | 1.99 | 0.90, 4.46 | 0.09 | 2.63 | 1.71, 4.04 | <0.01 | 2.59 | 1.47, 4.57 | <0.01 |
| Alpha blockers | 305 (2.80%) | | 1.24 | 0.92, 1.67 | 0.16 | 1.17 | 0.97, 1.41 | 0.09 | 0.51 | 0.37, 0.71 | <0.01 |
| Beta blockers | 265 (2.43%) | | 2.15 | 1.65, 2.81 | <0.01 | 1.76 | 1.48, 2.10 | <0.01 | 1.11 | 0.85, 1.46 | 0.43 |
| Statins | 339 (3.11%) | | 1.34 | 1.01, 1.77 | 0.04 | 0.93 | 0.76, 1.13 | 0.45 | 1.42 | 1.16, 1.73 | <0.01 |
| Vitamin D | 465 (4.27%) | | 1.63 | 1.29, 2.05 | <0.01 | 1.24 | 1.06, 1.46 | <0.01 | 1.52 | 1.27, 1.82 | <0.01 |

Table S2 – Unadjusted HRs for prognostic factors for men with localised disease associated with outcomes

HR – Hazard Ratio; BPH – Benign Prostatic Hypertrophy; COPD – Chronic Obstructive Pulmonary Disease; CVA – Cerebrovascular Accident; IHD – Ischaemic Heart Disease; PVD – Peripheral Vascular Disease; T2DM – Type 2 Diabetes Mellitus

For peer review only

| **n = 42, 182** | | | **Prostate cancer mortality** | | | **All-cause mortality** | | | **Systemic therapy** | | |
|---|---|---|---|---|---|---|---|---|---|---|---|
| **Factor** | **Mean (SD)** | **Missing [n (%)]** | **HR per SD** | **95% CI** | **p** | **HR per SD** | **95% CI** | **p** | **HR per SD** | **95% CI** | **p** |
| Age | 76.70 (9.42) | 0 | 1.55 | 1.49, 1.62 | <0.01 | 1.78 | 1.74, 1.82 | <0.01 | 0.67 | 0.65, 0.70 | <0.01 |
| BMI | 27.37 (4.49) | 394 (3.61%) | 0.96 | 0.92, 0.99 | 0.05 | 0.96 | 0.94, 0.98 | <0.01 | 1.06 | 1.02, 1.10 | <0.01 |
| Triglycerides | 1.48 (0.82) | 3,856 (35.37%) | 0.92 | 0.87, 0.99 | 0.02 | 0.95 | 0.91, 0.98 | <0.01 | 0.96 | 0.91, 1.00 | 0.06 |
| HDL cholesterol | 1.34 (0.53) | 3,954 (36.27%) | 1 | 0.94, 1.07 | 0.60 | 1 | 0.97, 1.04 | 0.80 | 1.03 | 0.99, 1.06 | 0.12 |
| LDL cholesterol | 2.84 (0.99) | 4,698 (43.10%) | 0.93 | 0.86, 0.99 | 0.03 | 0.89 | 0.86, 0.93 | <0.01 | 1.05 | 1, 1.10 | 0.03 |
| Hb | 141.81 (16.58) | 2,696 (24.73%) | 0.61 | 0.58, 0.63 | <0.01 | 0.66 | 0.64, 0.67 | <0.01 | 1.07 | 1.02, 1.13 | <0.01 |
| Albumin | 41.41 (4.31) | 2,954 (27.10%) | 1.71 | 0.99, 2.96 | 0.06 | 1.33 | 0.91, 1.94 | 0.15 | 0.75 | 0.46, 1.21 | 0.24 |
| Random glucose | 5.92 (2.11) | 4,525 (41.51%) | 1.06 | 1.01, 1.10 | 0.01 | 1.07 | 1.04, 1.09 | <0.01 | 0.92 | 0.87, 0.98 | 0.01 |
| | **Median (IQR)** | **Missing [n (%)]** | | | | | | | | | |
| PSA | 10.4 (6.11, 24.6) | 2,352 (21.58%) | 1.28 | 0.96, 1.71 | 0.10 | 0.84 | 0.73, 0.98 | 0.02 | 2.37 | 1.88, 2.99 | <0.01 |
| CRP | 5 (2, 10) | 8,061 (73.95%) | 2.50 | 1.03, 6.08 | 0.04 | 2.55 | 1.47, 4.42 | <0.01 | 1.03 | 0.51, 2.06 | 0.94 |
| Ferritin | 101.5 (45, 197) | 9,495 (87.10%) | 1.10 | 1.06, 1.15 | <0.01 | 1.04 | 0.99, 1.09 | 0.12 | 0.99 | 0.90, 1.09 | .089 |

Table S3 – Unadjusted HRs for prognostic factors for men with localised disease and unknown location associated with outcomes

HR – Hazard Ratio; SD – Standard Deviation; BMI – Body Mass Index; PSA – Prostate Specific Antigen; HDL – High Density Lipoprotein; LDL – Low Density Lipoprotein; HbA1c – Haemoglobin A1c; CRP – C-Reactive Protein; Hb – Haemoglobin

| n = 42, 182 | | | Prostate cancer mortality | | | All-cause mortality | | | Systemic therapy | | |
|---|---|---|---|---|---|---|---|---|---|---|---|
| **Factor** | **n (%)** | **Missing [n(%)]** | **HR** | **95% CI** | **p** | **HR** | **95% CI** | **p** | **HR** | **95% CI** | **p** |
| Smoker (current/ ex-) | 19,215 (45.56%) | 777 (7.13%) | 1.24 | 1.09, 1.40 | <0.01 | 1.36 | 1.26, 1.46 | <0.01 | 1.20 | 1.10, 1.30 | <0.01 |
| Excess alcohol | 5,926 (14.05%) | 4,370 (40.09%) | 0.75 | 0.62, 0.90 | <0.01 | 0.79[b] | 0.71, 0.88 | <0.01 | 1.01 | 0.90, 1.13 | 0.86 |
| BPH | 4,318 (10.24%) | 3,526 (32.35%) | 0.61 | 0.49, 0.76 | <0.01 | 0.79 | 0.70, 0.89 | <0.01 | 0.70 | 0.61, 0.80 | <0.01 |
| COPD | 3,866 (9.17%) | 3,583 (32.87%) | 0.95 | 0.77, 1.19 | 0.66 | 1.41 | 1.26, 1.58 | <0.01 | 1.08 | 0.94, 1.25 | 0.26 |
| CVA | 2,973 (7.05%) | 3,584 (32.88%) | 1.48 | 1.19, 1.85 | <0.01 | 1.89 | 1.67, 2.12 | <0.01 | 0.78 | 0.64, 0.96 | 0.02 |
| IHD | 7,512 (17.81%) | 3,405 (31.24%) | 1.62 | 1.39, 1.88 | <0.01 | 1.79 | 1.64, 1.95 | <0.01 | 0.89 | 0.79, 1.00 | 0.05 |
| PVD | 1,138 (2.70%) | 3,582 (32.86%) | 1.8 | 1.31, 2.49 | <0.01 | 1.89 | 1.55, 2.25 | <0.01 | 0.84 | 0.62, 1.14 | 0.26 |
| T2DM | 6,233 (14.78%) | 3,448 (31.63%) | 1.13 | 0.96, 1.33 | 0.16 | 1.04 | 0.94, 1.14 | 0.49 | 0.93 | 0.83, 1.04 | 0.20 |
| Aspirin | 2,022 (4.79%) | 16 (0.15%) | 2.33 | 1.88, 2.90 | <0.01 | 2.18 | 1.90, 2.49 | <0.01 | 1.24 | 0.99, 1.54 | 0.06 |
| Metformin | 220 (0.52%) | | 1.99 | 0.90, 4.46 | 0.09 | 2.63 | 1.71, 4.04 | <0.01 | 2.59 | 1.47, 4.57 | <0.01 |
| Alpha blockers | 1,025 (2.43%) | | 1.24 | 0.92, 1.67 | 0.16 | 1.17 | 0.98, 1.41 | 0.09 | 0.51 | 0.37, 0.71 | <0.01 |
| Beta blockers | 1,127 (2.67%) | | 2.15 | 1.65, 2.81 | <0.01 | 1.76 | 1.48, 2.10 | <0.01 | 1.11 | 0.85, 1.46 | 0.43 |
| Statins | 1,299 (3.08%) | | 1.34 | 1.01, 1.77 | 0.04 | 0.93 | 0.78, 1.13 | 0.45 | 1.42 | 1.16, 1.73 | <0.01 |
| Vitamin D | 2,093 (4.96%) | | 1.63 | 1.29, 2.05 | <0.01 | 1.24 | 1.06, 1.46 | 0.01 | 1.52 | 1.27, 1.88 | <0.01 |

Table S4 – Unadjusted HRs for prognostic factors for men with localised disease and unknown location associated with outcomes

HR – Hazard Ratio; BPH – Benign Prostatic Hypertrophy; COPD – Chronic Obstructive Pulmonary Disease; CVA – Cerebrovascular Accident; IHD – Ischaemic Heart Disease; PVD – Peripheral Vascular Disease; T2DM – Type 2 Diabetes Mellitus

For peer review only

| **n = 42, 182** | | | **Prostate cancer mortality** | | | **All-cause mortality** | | | **Systemic therapy** | | |
|---|---|---|---|---|---|---|---|---|---|---|---|
| **Factor** | **Mean (SD)** | **Missing [n (%)]** | **HR per SD[a]** | **95% CI** | **p** | **HR per SD[a]** | **95% CI** | **p** | **HR per SD[a]** | **95% CI** | **p** |
| Age | 76.70 (9.42) | 0 | 1.82 | 1.54, 2.16 | <0.01 | 2.00 | 1.84, 2.19 | <0.01 | 0.96 | 0.90, 1.01 | 0.01 |
| BMI | 27.37 (4.49) | 394 (3.61%) | 1.07 | 0.93, 1.23 | 0.36 | 0.96 | 0.89, 1.03 | 0.28 | 1.04 | 0.99, 1.09 | 0.10 |
| Triglycerides | 1.48 (0.82) | 3,856 (35.37%) | 0.89 | 0.70, 1.12 | 0.31 | 1.02[b] | 0.92, 1.13 | 0.72 | 1.03 | 0.97, 1.09 | 0.38 |
| HDL cholesterol | 1.34 (0.53) | 3,954 (36.27%) | 1.04 | 0.88, 1.23 | 0.67 | 0.97[b] | 0.86, 1.09 | 0.58 | 1.01 | 0.95, 1.07 | 0.79 |
| LDL cholesterol | 2.84 (0.99) | 4,698 (43.10%) | 0.84 | 0.68, 1.03 | 0.09 | 0.90[b] | 0.82, 1.01 | 0.05 | 0.99 | 0.94, 1.05 | 0.81 |
| Hb | 141.81 (16.58) | 2,696 (24.73%) | 0.71 | 0.59, 0.85 | <0.01 | 0.74 | 0.67, 0.80 | <0.01 | 0.90 | 0.85, 0.96 | <0.01 |
| Albumin | 41.41 (4.31) | 2,954 (27.10%) | 0.76 | 0.65, 0.89 | <0.01 | 0.81 | 0.75, 0.88 | <0.01 | 0.92 | 0.87, 0.97 | <0.01 |
| Random glucose | 5.92 (2.11) | 4,525 (41.51%) | 1.28 | 1.08, 1.53 | <0.01 | 1.11 | 0.99, 1.24 | 0.07 | 1.02[b] | 0.95, 1.09 | 0.58 |
| | **Median (IQR)** | **Missing [n (%)]** | | | | | | | | | |
| PSA | 10.4 (6.11, 24.6) | 2,352 (21.58%) | 1.19 | 1.04, 1.35 | 0.01 | 1.14 | 1.02, 1.28 | 0.02 | 1.09 | 0.92, 1.30 | 0.33 |
| CRP | 5 (2, 10) | 8,061 (73.95%) | 1.41 [b] | 1.12, 1.77 | <0.01 | 1.28[b] | 1.11, 1.47 | <0.01 | 1.07 | 0.95, 1.20 | 0.25 |
| Ferritin | 101.5 (45, 197) | 9,495 (87.10%) | 1.80 | 1.08, 3.00 | 0.02 | 1.02[b] | 0.67, 1.56 | 0.92 | 1.06 | 0.85, 1.32 | 0.62 |

Table S5 – Prognostic factors for men with localised disease and unknown location associated with outcomes

[a] Adjusted for age, PSA, Gleason score, TNM stage

[b] Proportional Hazards assumption test not met

HR – Hazard Ratio; SD – Standard Deviation; BMI – Body Mass Index; PSA – Prostate Specific Antigen; HDL – High Density Lipoprotein; LDL – Low Density Lipoprotein; HbA1c – Haemoglobin A1c; CRP – C-Reactive Protein; Hb – Haemoglobin

For peer review only

| n = 42, 182 | | | Prostate cancer mortality | | | All-cause mortality | | | Systemic therapy | | |
|---|---|---|---|---|---|---|---|---|---|---|---|
| **Factor** | **n (%)** | **Missing [n(%)]** | **HR[a]** | **95% CI** | **p** | **HR[a]** | **95% CI** | **p** | **HR[a]** | **95% CI** | **p** |
| Smoker (current/ ex-) | 19,215 (45.56%) | 777 (7.13%) | 1.41 | 1.04, 1.90 | 0.03 | 1.67 | 1.43, 1.96 | <0.01 | 1.22 | 1.10, 1.34 | <0.01 |
| Excess alcohol | 5,926 (14.05%) | 4,370 (40.09%) | 0.65 | 0.40, 1.05 | 0.08 | 0.89[b] | 0.71, 1.13 | 0.35 | 0.99 | 0.86, 1.13 | 0.85 |
| BPH | 4,318 (10.24%) | 3,526 (32.35%) | 0.63 | 0.38, 1.05 | 0.08 | 0.82 | 0.65, 1.03 | 0.08 | 0.73 | 0.62, 0.86 | <0.01 |
| COPD | 3,866 (9.17%) | 3,583 (32.87%) | 0.99 | 0.58, 1.66 | 0.96 | 1.68 | 1.35, 2.09 | <0.01 | 1.18 | 0.99, 1.40 | 0.06 |
| CVA | 2,973 (7.05%) | 3,584 (32.88%) | 0.64 | 0.30, 1.38 | 0.26 | 1.47 | 1.12, 1.93 | <0.01 | 0.90 | 0.71, 1.15 | 0.41 |
| IHD | 7,512 (17.81%) | 3,405 (31.24%) | 1.70 | 1.19, 2.44 | <0.01 | 1.31 | 1.08, 1.58 | <0.01 | 1.01 | 0.87, 1.00 | 0.9 |
| PVD | 1,138 (2.70%) | 3,582 (32.86%) | 2.52 | 1.23, 5.16 | 0.01 | 1.91 | 1.27, 2.85 | <0.01 | 1.09 | 0.75, 1.59 | 0.64 |
| T2DM | 6,233 (14.78%) | 3,448 (31.63%) | 1.06 | 0.72, 1.58 | 0.76 | 0.97 | 0.79, 1.19 | 0.75 | 1.02 | 0.88, 1.17 | 0.83 |
| Aspirin | 2,022 (4.79%) | 16 (0.15%) | 1.55 | 0.79, 3.02 | 0.20 | 1.41 | 0.99, 2.00 | 0.06 | 1.23 | 0.95, 1.60 | 0.12 |
| Metformin | 220 (0.52%) | | | | | 2.76 | 1.03, 7.38 | 0.04 | 1.43 | 0.64, 3.20 | 0.38 |
| Alpha blockers | 1,025 (2.43%) | | 1.28 | 0.59, 2.75 | 0.53 | 1.19 | 0.85, 1.67 | 0.32 | 0.55 | 0.38, 0.79 | <0.01 |
| Beta blockers | 1,127 (2.67%) | | 1.76 | 0.82, 3.75 | 0.15 | 1.82 | 1.27, 2.62 | <0.01 | 1.43 | 1.06, 1.93 | 0.02 |
| Statins | 1,299 (3.08%) | | 1.42 | 0.75, 2.69 | 0.28 | 0.87 | 0.57, 1.32 | 0.51 | 1.09 | 0.86, 1.37 | 0.48 |
| Vitamin D | 2,093 (4.96%) | | 1.13 | 0.55, 2.30 | 0.74 | 1.13 | 0.78, 1.65 | 0.51 | 1.38 | 1.12, 1.72 | <0.01 |

Table S6 – Prognostic factors for men with localised disease and unknown location associated with outcomes

[a] Adjusted for age, PSA, Gleason score, TNM stage

[b] Proportional Hazards assumption test not met

HR – Hazard Ratio; BPH – Benign Prostatic Hypertrophy; COPD – Chronic Obstructive Pulmonary Disease; CVA – Cerebrovascular Accident; IHD – Ischaemic Heart Disease; PVD – Peripheral Vascular Disease; T2DM – Type 2 Diabetes Mellitus

For peer review only

# TRIPOD Checklist: Prediction Model Development and Validation

| Section/Topic | Item | | Checklist Item | Page |
|---|---|---|---|---|
| **Title and abstract** | | | | |
| Title | 1 | D;V | Identify the study as developing and/or validating a multivariable prediction model, the target population, and the outcome to be predicted. | 1 |
| Abstract | 2 | D;V | Provide a summary of objectives, study design, setting, participants, sample size, predictors, outcome, statistical analysis, results, and conclusions. | 3 |
| **Introduction** | | | | |
| Background and objectives | 3a | D;V | Explain the medical context (including whether diagnostic or prognostic) and rationale for developing or validating the multivariable prediction model, including references to existing models. | 4-5 |
| | 3b | D;V | Specify the objectives, including whether the study describes the development or validation of the model or both. | 3, 5 |
| **Methods** | | | | |
| Source of data | 4a | D;V | Describe the study design or source of data (e.g., randomized trial, cohort, or registry data), separately for the development and validation data sets, if applicable. | 6 |
| | 4b | D;V | Specify the key study dates, including start of accrual; end of accrual; and, if applicable, end of follow-up. | 6 |
| Participants | 5a | D;V | Specify key elements of the study setting (e.g., primary care, secondary care, general population) including number and location of centres. | 6 |
| | 5b | D;V | Describe eligibility criteria for participants. | 6 |
| | 5c | D;V | Give details of treatments received, if relevant. | 6 |
| Outcome | 6a | D;V | Clearly define the outcome that is predicted by the prediction model, including how and when assessed. | 6 |
| | 6b | D;V | Report any actions to blind assessment of the outcome to be predicted. | n/a |
| Predictors | 7a | D;V | Clearly define all predictors used in developing the multivariable prediction model, including how and when they were measured. | 6, 8 |
| | 7b | D;V | Report any actions to blind assessment of predictors for the outcome and other predictors. | n/a |
| Sample size | 8 | D;V | Explain how the study size was arrived at. | 7 |
| Missing data | 9 | D;V | Describe how missing data were handled (e.g., complete-case analysis, single imputation, multiple imputation) with details of any imputation method. | 6-7 |
| Statistical analysis methods | 10a | D | Describe how predictors were handled in the analyses. | 6-7 |
| | 10b | D | Specify type of model, all model-building procedures (including any predictor selection), and method for internal validation. | 6-7 |
| | 10c | V | For validation, describe how the predictions were calculated. | n/a |
| | 10d | D;V | Specify all measures used to assess model performance and, if relevant, to compare multiple models. | n/a |
| | 10e | V | Describe any model updating (e.g., recalibration) arising from the validation, if done. | n/a |
| Risk groups | 11 | D;V | Provide details on how risk groups were created, if done. | n/a |
| Development vs. validation | 12 | V | For validation, identify any differences from the development data in setting, eligibility criteria, outcome, and predictors. | n/a |
| **Results** | | | | |
| Participants | 13a | D;V | Describe the flow of participants through the study, including the number of participants with and without the outcome and, if applicable, a summary of the follow-up time. A diagram may be helpful. | 8 |
| | 13b | D;V | Describe the characteristics of the participants (basic demographics, clinical features, available predictors), including the number of participants with missing data for predictors and outcome. | 8, Table 1 |
| | 13c | V | For validation, show a comparison with the development data of the distribution of important variables (demographics, predictors and outcome). | n/a |
| Model development | 14a | D | Specify the number of participants and outcome events in each analysis. | 8 |
| | 14b | D | If done, report the unadjusted association between each candidate predictor and outcome. | Table S1-2 |
| Model specification | 15a | D | Present the full prediction model to allow predictions for individuals (i.e., all regression coefficients, and model intercept or baseline survival at a given time point). | n/a |
| | 15b | D | Explain how to use the prediction model. | n/a |
| Model performance | 16 | D;V | Report performance measures (with CIs) for the prediction model. | n/a |
| Model-updating | 17 | V | If done, report the results from any model updating (i.e., model specification, model performance). | n/a |
| **Discussion** | | | | |
| Limitations | 18 | D;V | Discuss any limitations of the study (such as nonrepresentative sample, few events per predictor, missing data). | 11 |
| Interpretation | 19a | V | For validation, discuss the results with reference to performance in the development data, and any other validation data. | n/a |
| | 19b | D;V | Give an overall interpretation of the results, considering objectives, limitations, results from similar studies, and other relevant evidence. | 9-12 |
| Implications | 20 | D;V | Discuss the potential clinical use of the model and implications for future research. | 12 |
| **Other information** | | | | |
| Supplementary information | 21 | D;V | Provide information about the availability of supplementary resources, such as study protocol, Web calculator, and data sets. | 6 |
| Funding | 22 | D;V | Give the source of funding and the role of the funders for the present study. | 2 |

*Items relevant only to the development of a prediction model are denoted by D, items relating solely to a validation of a prediction model are denoted by V, and items relating to both are denoted D;V. We recommend using the TRIPOD Checklist in conjunction with the TRIPOD Explanation and Elaboration document.

For peer review only
